# Improving Antimicrobial Activity and Physico-Chemical Properties by Isosteric Replacement of 2-Aminothiazole with 2-Aminooxazole

**DOI:** 10.3390/ph15050580

**Published:** 2022-05-06

**Authors:** Martin Juhás, Andrea Bachtíková, Daria Elżbieta Nawrot, Paulína Hatoková, Vinod Sukanth Kumar Pallabothula, Adéla Diepoltová, Ondřej Janďourek, Pavel Bárta, Klára Konečná, Pavla Paterová, Vít Šesták, Jan Zitko

**Affiliations:** 1 Faculty of Pharmacy in Hradec Králové, Charles University, Akademika Heyrovského 1203, 500 05 Hradec Králové, Czech Republic; bachtikand@faf.cuni.cz (A.B.); nawrotd@faf.cuni.cz (D.E.N.); hatokovp@faf.cuni.cz (P.H.); pallabov@faf.cuni.cz (V.S.K.P.); diepolta@faf.cuni.cz (A.D.); jando6aa@faf.cuni.cz (O.J.); pavel.barta@faf.cuni.cz (P.B.); konecna@faf.cuni.cz (K.K.); 2 Department of Clinical Microbiology, University Hospital Hradec Králové, Sokolská 581, 500 05 Hradec Králové, Czech Republic; pavla.paterova@fnhk.cz; 3 Department of Clinical Biochemistry and Diagnostics, Faculty of Medicine in Hradec Králové, University Hospital, Sokolská 581, 500 05 Hradec Králové, Czech Republic; vit.sestak@fnhk.cz

**Keywords:** aminooxazole, aminothiazole, antimycobacterial activity, docking, molecular docking, molecular dynamics, isostere, pyridine, water solubility

## Abstract

Antimicrobial drug resistance is currently one of the most critical health issues. Pathogens resistant to last-resort antibiotics are increasing, and very few effective antibacterial agents have been introduced in recent years. The promising drug candidates are often discontinued in the primary stages of the drug discovery pipeline due to their unspecific reactivity (PAINS), toxicity, insufficient stability, or low water solubility. In this work, we investigated a series of substituted *N*-oxazolyl- and *N*-thiazolylcarboxamides of various pyridinecarboxylic acids. Final compounds were tested against several microbial species. In general, oxazole-containing compounds showed high activity against mycobacteria, especially *Mycobacterium tuberculosis* (best MIC_H37Ra_ = 3.13 µg/mL), including the multidrug-resistant strains. Promising activities against various bacterial and fungal strains were also observed. None of the compounds was significantly cytotoxic against the HepG2 cell line. Experimental measurement of lipophilicity parameter log k’_w_ and water solubility (log *S*) confirmed significantly (typically two orders in logarithmic scale) increased hydrophilicity/water solubility of oxazole derivatives in comparison with their thiazole isosteres. Mycobacterial β-ketoacyl-acyl carrier protein synthase III (FabH) was suggested as a probable target by molecular docking and molecular dynamics simulations.

## 1. Introduction

Drug resistance is a huge limitation in the treatment of deadly diseases caused by mycobacteria and bacteria both in hospitals and community-wide [1]. Until the emergence of SARS-CoV-2, tuberculosis (TB) was the leading cause of fatality in humans caused by a single infectious agent—*Mycobacterium tuberculosis* (Mtb). The World Health Organization (WHO) reports that TB was responsible for more than 1.5 million deaths in 2020, which is an increase from 1.4 million reported in 2019 [2,3]. Furthermore, 1.7 billion people, almost one-fourth of the global population, are estimated to be currently infected by Mtb but are in the dormant stage (also referred to as latent tuberculosis infection) [2]. Considering the financial burden and common occurrence of drug resistance during the usual 6-month regimen (in non-complicated cases), the numbers are alarming. Currently, there are three major types of drug-resistant TB: rifampicin-resistant (RR-TB), multi-drug-resistant (MDR-TB), and extensively drug-resistant (XDR-TB) [2,4]. Yet, anti-TB drug development has been at its all-time low since 2008, and only a few new drug candidates are in the clinical stages. New drugs are therefore needed [5].

Other pathogens of huge worldwide significance include so-called atypical mycobacteria (*M. avium*) but also bacterial species known under the abbreviation ESKAPE (*Enterococcus faecium*, *Staphylococcus aureus*, *Klebsiella pneumoniae*, *Acinetobacter* *baumannii*, *Pseudomonas aeruginosa*, and *Enterobacter* species). These mostly nosocomial pathogens display multi-drug or even pan-drug resistance associated with high mortality and are therefore categorized by the WHO among 12 bacteria against which new antibiotic drugs are urgently needed [6]. Unfortunately, investments needed in this area would not be sufficiently profit-making, which discourages pharmaceutical companies and thus leads to a decline in the development of new antimicrobials. However, the drug resistance among clinical isolates is rising and may set humanity back to the pre-antibiotic era [1,7].

### 1.1. Aminothiazoles in Antimicrobial Agents

Among five-membered heterocycles, thiazole, and especially 2-aminothiazole (2-AMT), has a privileged position in antimicrobial research and is contained in many highly active compounds [8,9,10,11]. Several successful marketed drugs can already be identified as bearing the 2-AMT fragment, e.g., aztreonam or the whole 3rd generation of cephalosporins, where the 2-AMT fragment is a determining sign (e.g., cefotaxime). Despite its apparent success, 2-AMT is considered very problematic for drug design due to often observed unspecific reactivity or promiscuous behavior. The presence of sulfur infers the possibility of undesirable interactions with the GSH system or unwanted biotransformation, e.g., S-oxidation or oxidative ring openings [12,13]. As a consequence, several 2-AMT-containing groups are currently categorized as PAINS and thus often disqualified in the discovery of new chemical entities [14,15].

Isosteric replacement of 2-AMT with 2-aminooxazole (2-AMO) is a common medicinal chemistry practice [16], but it does not seem to be as extensively established in the design of antimicrobials. Recently, Azzali and colleagues [8] have pointed out that 2-AMO could potentially alleviate at least some of the often-observed negatives of 2-AMT (described above) and showed that the 2-AMO isosteres behaved correspondingly to their 2-AMT counterparts in terms of antimicrobial activity, while not presenting any significant cytotoxicity or PAINS-like behavior. Even though Azzali et al. investigated this substitution in the context of antimycobacterial agents, we see no apparent reason why it could not also be translated to other antimicrobial areas as already seen in the literature, e.g., in references [17,18]. Currently, only one 2-AMO-containing antimicrobial drug is marketed in the world—sulfonamide derivative sulfamoxole (not approved by the FDA)—but several promising 2-AMO derivatives have already been identified as the inhibitors of bacterial biotin carboxylase [19] or the derivatives active against drug-resistant Mtb strains [20].

### 1.2. Design of the Compounds

**Figure 1:**
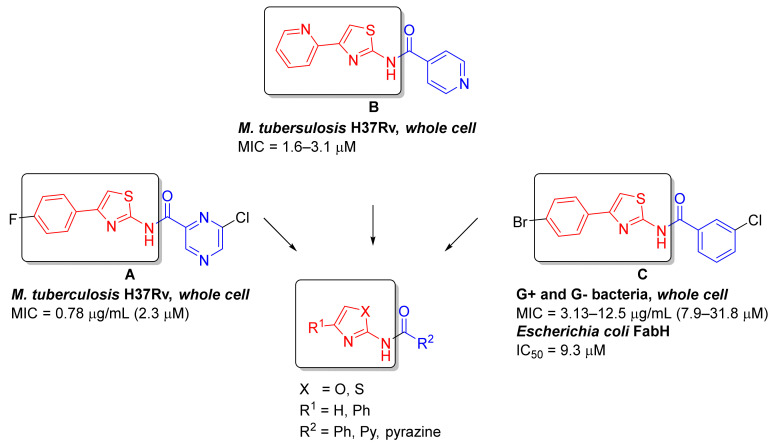
Design rationale and general structure of the investigated derivatives. All MIC values were recalculated to µM. References: **A** [11], **B** [21], **C** [9].

Like other groups researching antimicrobial compounds, we have also encountered issues with 2-AMT in otherwise highly active compounds. In some derivatives, the issues with low solubility led to the inability to carry on with antibacterial evaluations, e.g., by Zitko and colleagues [11]. Inspired by the results of Azzali et al. [8], we decided to test the hypothesis of improvement of physico-chemical properties while retaining antimicrobial activities for the 2-AMT -> 2-AMO isosteres. Title compounds were designed as a pivotal series of heteroaromatic derivatives of the general structure presented in Figure 1 based on the known antimicrobial (antibacterial and antimycobacterial) agents and FabH inhibitors, especially acyl derivatives described by Zitko and colleagues [11] (structure **A** in Figure 1), Meissner and colleagues [21] (structure **B**), and Li and colleagues [9] (structure **C**). The FabH enzyme (β-ketoacyl-acyl carrier protein synthase III, or KAS III) represents a very promising antimicrobial target involved in the synthesis of fatty acids through initiation of the FAS II cycle that is not present in humans [22].

In this study, we focused only on non-substituted and 4-phenyl-substituted 2-AMT and 2-AMO as it allowed easier determination of the oxazole effect (R^1^ in Figure 1). Other substitutions will be evaluated in upcoming studies. Acidic (aroyl) moieties (R^2^ in Figure 1) were chosen to represent the most common aromatic and heteroaromatic cores investigated in antimicrobial agents as referenced previously or as investigated, e.g., by Nawrot and colleagues [23].

Synthesized derivatives were evaluated for antimicrobial activity against a palette of (myco)bacteria and fungi and for their cytotoxicity (HepG2 cells were used). Physico-chemical properties such as solubility and lipophilicity were also examined.

## 2. Results and Discussion

### 2.1. Synthesis and Analytical Evaluation

**Figure 2:**
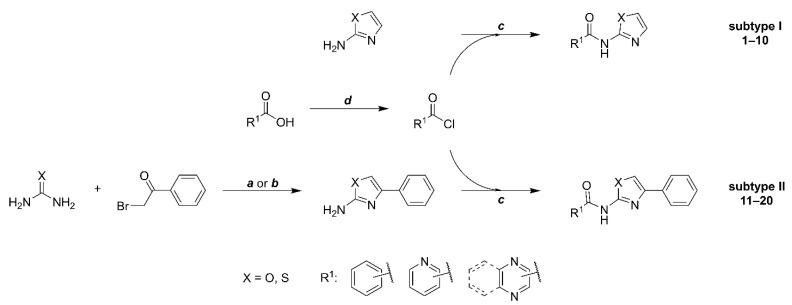
Synthetic procedure used to prepare title compounds. Conditions: **a:** (for X = S) 1.1 eq. urea, in EtOH, reflux 2 h; **b:** (for X = O) 10 eq. urea, in MeCN, reflux 16 h or in DMF, 120 °C 2 h; **c**: 1 eq. acyl chloride, 3 eq. DIPEA or pyridine, in DCM, overnight; **d**: 10 eq. thionyl chloride, catalytic DMF.

The title compounds (Table 1) were prepared using conventional procedures, as exemplified in Figure 2. Details can be found in the *Materials and Methods*, Section 3.

Non-substituted 2-AMO/2-AMT used for subtype I compounds were obtained from commercial sources and used without further purification. The 2-AMO/2-AMT fragment of subtype II (4-phenyl-substituted 2-AMO/2-AMT) was prepared by reacting 2-bromoketone with thiourea (method **a** [9] in Figure 2) or urea (method **b** [8]) as described before. For the final coupling, we have investigated several procedures, including coupling reagents such as 1,1′-carbonyldiimidazole (CDI). As before [24], CDI coupling was successful for the derivatives containing the 2-AMT but failed in all syntheses involving 2-AMO and was thus discontinued in favor of acylation by acyl chlorides. In general, we observed low to moderate final yields for the majority of compounds. Yields were lower in 2-AMO-containing derivatives due to the overall low nucleophilicity of the 2-amino group compared to 2-AMT. However, since the gathered amounts and the obtained purity of final compounds were sufficient for all subsequent biological and analytical screenings, we did not attempt to improve it further. The samples of compounds **18a** and **19a** were identical to those used in the original study [11].

The analytical data of all synthesized compounds and intermediates agreed with the proposed structures based on ^1^H- and ^13^C-NMR, IR, and HR-MS analyses. The ^1^H-NMR spectra clearly showed signals indicating the presence of the carboxamide and the thiazole or oxazole ring. In subtype I, the carboxamide hydrogen signal ranged from 13.06 to 11.41 ppm and appeared as a broad singlet. The thiazole hydrogens were observed as two doublets, one between 7.59 and 7.55 ppm, the second from 7.39–7.29 ppm (*J* = 3.6 Hz). The oxazole hydrogens, also seen as two doublets, appeared from 7.99–7.75 ppm and 7.27–7.17 ppm (*J* = 1.0 Hz). In subtype II derivatives, the carboxamide hydrogen signal appeared as a singlet, usually between 13.16 and 10.86 ppm. In subtype II compounds, the thiazole hydrogen appeared as a singlet at 7.88–7.21 ppm, while the oxazole hydrogen was present as a singlet at 8.51–8.21 ppm (in deuterated DMSO). In some compounds, not all carbon signals were visible on ^13^C-NMR, which can be explained by signals merging due to similar ppm values. Representative NMR spectra of subtype I and II compounds may be seen in the Supplementary Materials.

The strong carbonyl stretching of carboxamide C=O in the 2-AMO derivatives was mostly observed at approx. 1700 cm^−1^, while in 2-AMT we recorded it at a slightly lower value around 1675 cm^−1^ for both subtype I and subtype II compounds. The C-H stretching of (hetero)aromates was recorded at approx. 3100 cm^−1^, the methyl groups were recorded at 2920 cm^−1^. The phenyl C=C stretching appeared at approx. 1630 to 1400 cm^−1^.

The purity of the final derivatives used for the biological evaluations was >97% (except compound **17b** with 95%), as analyzed by the UHPLC. Mass accuracy deviation in the HR-MS identification did not exceed ±2.06 ppm.

### 2.2. Investigation of Lipophilicity

Lipophilicity and water solubility of the drug candidates are important parameters as they are often directly related to their ADME properties in the organism [25]. Lipophilicity is usually expressed as the logarithm of the partition coefficient in *n*-octanol/water (log *P*). Due to its laborious determination, especially in the HTS screening, other experimental methods such as RP-HPLC log k’_w_ [26] or purely computational approaches such as Clog *P* were proposed and have been established as reasonable estimators of true lipophilicity. To investigate the changes in lipophilicity upon introduction of oxazole in the title compounds, we determined the log k’_w_ of all derivatives and compared respective values among the pairs. All results are summarized in Table 1.

The isosteric exchange of thiazole for oxazole was associated with a decrease in log k’_w_ by 0.95 ± 0.09 (8.87 ± 1.22 times, n = 8) in subtype I and 1.05 ± 0.04 (11.33 ± 1.09 times, n = 6) in subtype II. The additional phenyl substituent in subtype II increased the log k’_w_ by 1.03 ± 0.13 (10.76 ± 1.36, n = 7) for the 2-aminothiazoles and by 0.89 ± 0.23 (7.70 ± 1.69, n = 7) for the 2-aminooxazoles. The experimental value of log k’_w_ linearly correlated with the log *P* calculated by SILICOS-IT software employing a hybrid fragmental/topological method (freely available in SwissADME [27]), $\log\;P=1.1423\;\log\;k{’}_{w}+0.5072\;\left(R^{2}=0.8368\right)$ . For the correlation plot, see Figure S1 in the Supplementary Materials.

Drug-likeness qualitatively estimates the chance for a molecule to become an oral drug with respect to its bioavailability. To evaluate our compounds in this regard, we used the SwissADME tool [27], which implements various established filters and computational algorithms, including the famous Lipinski [28], or newer ones, e.g., Veber [29] or Muegge [30] criteria to describe the drug-likeness of candidate molecules. All title compounds were “drug-like” based on all investigated criteria, except four compounds with MW < 200 (**1b**, **2b**, **3b**, **8b**) that did not fulfill the molecular weight criterion of Muegge (200 ≤ MW ≤ 600). Additionally, all of our compounds were evaluated as highly absorptive based on the boiled-egg method, also implemented in SwissADME [31].

### 2.3. Investigation of the Water Solubility

A low value of log *P* is often presumed to be related to increased water solubility, although this might not always be the case since log *P* is a measure of relative solubility of the compound in the lipophilic and hydrophilic solvents (at equilibrium). Therefore, to be sure, we tested whether the lowered log k’_w_ (related to log *P*) of the oxazole derivatives would also translate to increased solubility in water in a few representative derivatives. The water solubility of selected compounds was assessed by the kinetic method based on water-induced precipitation of DMSO stock solutions of tested compounds due to reasons described by Azzali and colleagues [8].

The evaluation started with the pair of the most active compounds **6a** and **6b** from subtype I. We were able to measure the solubility of **6a** (see Table 2), but **6b** did not precipitate after dilution of the DMSO stock solution with water, so it was not possible to determine its solubility. We thus presume that oxazole-containing derivative **6b** is more soluble in water (solubility > 500 μM) than **6a**. This is consistent with the calculated log *S* obtained from SwissADME (see below in Table 2). The second measured pair representing structurally similar active compounds of subtype II was **15a** and **15b**. Once again, better solubility (59 times) was observed in the 2-aminooxazole derivative **15b**. Additionally, better solubility of 2-aminooxazoles was also confirmed in the pair **12a** and **12b**.

Reasonable agreement of the experimentally determined solubility, expressed as log *S,* was noted with the calculated log *S* predicted based on Ali and colleagues [32] relying on topographical polar surface area (TPSA, the calculation also implemented in SwissADME), as seen in Table 2. Due to laborious experimental determination of the solubility of our compounds, we propose this model to be an acceptable predictor. However, we are aware that significantly more log *S* values would need to be experimentally determined and compared to the calculated ones to comment on statistics or goodness of prediction, which is beyond our intentions.

### 2.4. Antimicrobial Results

#### 2.4.1. In Vitro Screening of Antimycobacterial Activity

In vitro antimycobacterial activity of synthesized derivatives was determined by microplate Alamar Blue assay (MABA [33]) on *Mycobacterium tuberculosis* H37Ra ITM-M006710 (ATCC 9431), rapidly growing *M. smegmatis* DSM 43465 (ATCC 607), *M. aurum* DSM 43999 (ATCC 23366), and non-tuberculous mycobacteria *M. avium* DSM 44156 (ATCC 25291), *M. kansasii* DSM 44162 (ATCC 12478). Best selected compounds were also tested against Mtb H37Rv CNCTC My 331/88 (ATCC 27294) and multi-drug-resistant (MDR) clinical isolates of Mtb. The minimum inhibitory concentration (MIC) values were measured in µg/mL. The fast-growing *M. smegmatis* and *M. aurum* [34] (recently reclassified as genus *Mycolicibacteria* [35]) are commonly used as valid and safe non-pathogenic models for antimycobacterial research as an alternative to H37Rv [36,37,38].

#### 2.4.2. Comparing the Activity of 2-AMO with 2-AMT

Studying the potential of 2-AMO to replace 2-AMT in antimicrobial agents, we were curious if the activity within comparable pairs of the derivatives containing either core would differ. For convenient comparison, we coded the title compounds based on the presence of either core. All “**a**” codes are 2-AMT-containing derivatives, while the “**b**” code compounds always contain 2-AMO. Due to its relative clinical importance, the following discussion will be focused primarily on activity against Mtb (as tested on the surrogate avirulent strain H37Ra) unless stated otherwise, and the results can be seen in Table 1. Results of antimycobacterial activity against other strains can be found in Table S2 in the Supplementary Materials.

Ten out of 34 synthesized derivatives showed high antimycobacterial activity with MIC at or below 7.81 µg/mL, which we consider a reasonable activity cut-off. If the activity was present in a specific 2-AMT-containing derivative, the activity was always present in its 2-AMO counterpart. The difference in observed activity between 2-AMT and 2-AMO derivatives varied between studied subtypes. The antimycobacterial activity of 2-AMO-containing compounds of subtype I increased profoundly compared to the 2-AMT counterparts. As seen in Table 1, the 2-AMO derivative **6b**, which was also one of the most active compounds of the series, had MIC = 3.125 µg/mL, while its 2-AMT counterpart **6a** had no noticeable activity at the tested concentrations (MIC ≥ 500 µg/mL). A similar trend was observed for the 6-Me pair **7b** vs. **7a** and also in other AMO-AMT pairs of subtype I. The low observed antimycobacterial activity of the pyrazine derivative **8a** is consistent with the results already described by Doležal and colleagues [39] (compound 1, 0% at 6.25 µg/mL) and replacement of 2-AMT with 2-AMO in **8b** did not lead to noteworthy improvement (62.5 to 31.25 µg/mL).

In subtype II compounds (4-phenyl-substituted derivatives), the activities of 2-AMO and 2-AMT derivatives did not differ significantly, e.g., compounds **11a** and **11b** had the same MIC = 3.91 µg/mL, or **15a** and **15b** showed MIC = 3.91 and 7.81 µg/mL, respectively. The 2-AMT-containing compound appeared more active only in one pair, **13a** and **13b**. However, the difference was within the method-associated error, and the activity is thus comparable. Importantly, compound **17b**, which is a 2-AMO isostere of the derivative described by Zitko and colleagues (originally denoted 7a in [11]), showed increased solubility and higher activity against Mtb. Similarly, activity also improved upon the introduction of 2-AMO in its 5-Cl derivative (compound **18b**), in both cases reaching MIC = 15.625 µg/mL for the 2-AMO isosteres of previously published and inactive 2-AMT derivatives. For further results, see Table 1 and Table S2 in the Supplementary Materials.

#### 2.4.3. Structure–Activity Relationship

In general, pyridine derivatives were more active compared to other tested (hetero)aromates in our series, although pyrazines followed closely. The activity of benzene (**20b**) and quinoxaline derivatives was lower, with the latter being among the least active compounds of the series (except compound **10b**). Low activity of unsubstituted benzene derivative is in agreement with our discussion since even its thiazole counterpart has been found to be inactive, as described by Meissner (a different method was used, MIC_GAST_ or MIC_7H9-glucose_ ≥25 uM, originally compound 90 in reference [21]). The lower activity could be due to low water solubility and lower penetration compared to other compounds.

Comparing (unsubstituted) pyridine derivatives **11b**, **12b**, and **13b**, it seems that the heteroatom should be in position 2 or 4 relative to the carboxamide linker. The highest activity was reached with picolinic (**11b**, **11a**) and isonicotinic acids (**13a**) with MIC ranging from 3.91–7.81 µg/mL, which is up to a 20-fold increase in comparison to the other tested (hetero)aromatic cores. The nitrogen in position 3 had a rather activity-lowering effect (see, e.g., **11b** vs. **12b)**. The effects of moving the heteroatom to different positions were more noticeable in subtype I compounds than in subtype II. The addition of another heteroatom (pyridine to pyrazine) did not seem to add any benefit as in the case of **11b** vs. **17b**, yet no other positions nor heteroatoms other than nitrogen were tested in this study. Pyridine acids were also investigated previously by Meissner and colleagues [21] and were found to be less active than benzoic acid. However, they investigated a 4-(pyridin-2-yl)thiazole fragment, whereas we investigated 4-phenylthiazoles. The activity of a few other heterocycles against mycobacteria, where the compounds are structurally closely related to our 2-AMT series, can be seen in the literature, e.g., (nitro)furans [40] or quinolines [41], yet the data are still limited only to one heteroatom. Furthermore, there is no information about the activity of the 2-aminooxazole counterparts that we showed to be of high importance. Additional heteroatoms could greatly influence the acid–basic properties, impacting both pharmacokinetics and pharmacodynamic characteristics of the drug and should thus be investigated in further studies. Different activities arising from the position of heteroatoms could be tied to target interactions. However, as the exact mechanism of action as well as the binding mode are unknown, the differences could not be investigated as profoundly in this study.

Appropriate substitution has a crucial effect on the activity of drugs. We investigated common small substituents such as methyl or chlorine (based on commercial availability) in the aroyl part, as mentioned in the *Introduction*. In this study, no substitution was attempted in the 4-phenyl fragment. In general, the additional substitution of the heteroaroyl carboxamide led to increased activity as seen, e.g., for **3b** and **6b**. High activity was observed with chlorine in position 3 (relative to carboxamide) as the aforementioned **6b,** activity also increased with methyl substitution at the same position as in derivative **4b** or **5b**. Similar behavior was also observed in the previous studies of thiazole derivatives by Zitko and colleagues [11] or Meissner and colleagues [21]. This “meta-substitution” with chlorine or methyl increased the activity in both subtype I and II compounds containing both thiazole and oxazole cores. Other substitutions are currently under investigation, and once finished, we will be able to postulate more detailed structure–activity relationships in the oxazole series.

#### 2.4.4. Activity against Fast-Growing and Atypical Mycobacteria

Several compounds showed broad-spectrum activity and were highly active against all tested mycobacterial strains. Activity against *Mycobacterium avium* and *M. kansasii* is of particular importance as they are the agents most commonly responsible for opportunistic infections in immunocompromised patients [42,43]. Compounds **6b**, **7b**, **11b**, and **15b** were active against all five tested standard strains, and the observed activities were in the same range. This could mean that the target is not species-specific, which is a very desirable property for modern anti-TB drugs. We thus presume that more of the title compounds, if tested, would also show activity against the MDR strains of Mtb.

Interestingly, compound **19b,** which was inactive against Mtb, was strongly active against *M. avium*, which is intrinsically isoniazid-resistant, contributing to its hard-to-treat character. Reasons for this preference were not investigated further but could be of great significance. Activities of the title compounds against the tested fast-growing and atypical mycobacteria can be found in Table S2 in the Supplementary Materials.

#### 2.4.5. Activity against Mtb H37Rv and Multi-Drug-Resistant Clinical Isolates

The best active compounds **6b** and **7b** were further evaluated against virulent (Mtb H37Rv) and MDR clinical isolates of Mtb. The used MDR isolates were resistant to streptomycin, and almost all first-line antituberculars, namely isoniazid, rifampicin, and pyrazinamide, and were only sensitive to ethambutol. In all three tested strains, the antimycobacterial activity was comparable to the already presented activities against Mtb H37Ra and, most importantly, no decreased activity was observed in MDR strains, see Table 3. The presumed target in mycobacteria is thus unique, not related to any of the usual first-line antituberculars, that further promotes research of the most active derivatives in clinical applications. The complete resistance profile of the MDR strains can also be found in Table S1 in the Supplementary Materials.

The effects of isosteric replacement of 2-AMT for 2-AMO would be seen best in small, non-flexible molecules, where higher polarity and/or better solubility due to 2-AMO could not be masked by other effects, e.g., steric hindrance, intramolecular interactions, or overall low hydrophilicity. Compounds of subtype I fulfill these criteria, and the introduction of 2-AMO in the majority of derivatives led to a significant improvement of antimycobacterial activity compared to 2-AMT. In most cases, compounds went from non-actives to actives. Whether it was “just” the sufficient drug penetration through membranes as a result of improved physico-chemical properties or an interaction with the specific target was involved is unclear and needs to be verified further. However, the 2-AMO-related improvement of physico-chemical properties in subtype II compounds did not significantly alter the antimycobacterial potency. This strongly suggests that upon the introduction of 2-AMO in subtype I derivatives it was “just” the improved physico-chemical properties that led to the increased antimycobacterial activity and not any changes related to the drug target.

#### 2.4.6. In Vitro Screening of Antibacterial and Antifungal Activity

As mentioned in the *Introduction*, problems with solubility have already disqualified testing of some of our thiazole-containing derivatives. In this series, we observed only one compound that was insoluble in the testing medium for antibacterial and antifungal evaluation—**18b**. Activities of all other derivatives were tested on a set of sixteen microorganisms, eight bacteria and eight fungi. A microdilution broth method according to EUCAST [44,45,46] was used. MIC values were expressed in µM. Derivatives **12a** and **13a** were tested only up to 125 µM, **16b**, **19a** up to 250 µM. The rest were tested up to 500 µM. For the methodology and the complete list of tested strains, please see the Supplementary Materials.

Some compounds active against mycobacteria also showed potency against bacteria and fungi, yet the activity was weaker. Low activity against G+ bacteria was observed in compounds **6b**, **7b**, **15b**, and **16b**. Compounds **6b**, **7b** showed low antifungal activity against *Candida albicans*, *Lichtheimia corymbifera*, and *Trichophyton interdigitale*. Compound **9b** showed activity against *T. interdigitale*. In all cases, the best MIC was from 31.25 to 62.5 µM. The other compounds were inactive at tested concentrations (MIC above the highest tested concentration). For the full results of antibacterial and antifungal screening, see Table S3 and Table S4 in the Supplementary Materials**.**

Despite being weaker against bacteria and fungi, oxazoles **6b**, **7b**, **15b**, and **16b** showed significant broad-spectrum activity against different microorganisms, and further optimizations to target others are therefore highly encouraged. Importantly, thiazole counterparts of these compounds were inactive against bacteria and fungi. Since no apparent issues were noted (e.g., low solubility of the derivatives), this discrepancy could be safely attributed to the improved physico-chemical properties of the oxazole derivatives, probably resulting in better penetration through microbial membranes in comparison with the thiazoles, as seen in mycobacteria (see Section 2.4.1 *In Vitro Screening of Antimycobacterial Activity*).

### 2.5. In Silico Studies

#### 2.5.1. Docking

Due to the structural similarity of the title compounds to the described inhibitors of EcFabH synthesized by Li and colleagues [9] (compound **C** in Figure 1), we hypothesized MtFabH as a potential target of our compounds in mycobacteria.

We investigated the most likely binding mode of the title compounds in MtFabH using **6b** and **15b**, as representative examples of active compounds of both subtypes. The derivatives were docked to biologically relevant dimers of MtFabH (PDB ID: 1U6S) and poses were investigated in both subunits. The highest scoring poses were virtually identical in both active sites (subunits), only differing by the rotation of the chloropyridine core in **15b,** or by rotation of the oxazole ring in **6b** (no rotation of chloropyridine was observed). In the context of this study, the term “binding mode” is used to generalize the overall conformation of the ligand in the active site. It is presumed that despite different substituents, the overall pose of all active compounds should be similar. Therefore, the minute differences in poses described above were of no particular concern as the overall conformation of the molecule was identical, meaning both poses were considered as one binding mode.

**Figure 3:**
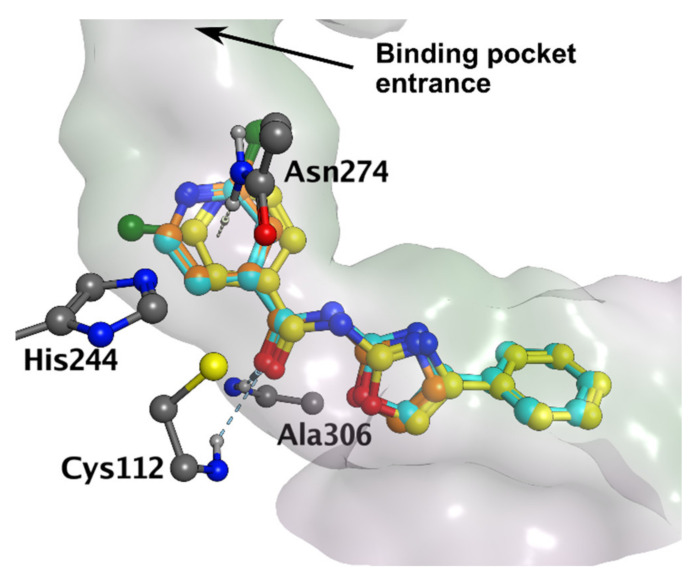
Binding mode 1 of **6b** (orange) and **15b** (yellow and cyan) in MtFabH (PDB ID: 1U6S), non-interacting hydrogens hidden for clarity.

The obtained binding mode matched the binding mode 1 reported by Zitko and colleagues [11] and will be referred to as such from now on. The ligand occupied the binding pocket near the catalytic triad (His244, Cys112, Asn274), and the aroyl ring pointed towards the entrance of the active site (“aroyl-out” mode, see Figure 3). In both **6b** and **15b**, we observed the same interactions with the receptor: hydrogen bonds to carboxamide oxygen originating from Cys112 and Ala306 (backbone NH in both cases) and the NH-π interactions [47] to pyridine core originating from the Asn274 sidechain carboxamide. No significant interactions of the phenyl substituent in **15b** were observed. The second binding mode (“aroyl-in”) described by Zitko as binding mode 2 was also observed, but the score was significantly lower (−6.6 vs. −8.1). Description of binding mode 1, the scores, and the energies of the interactions as calculated by the used force field are presented in Table 4.

We also investigated whether the apparent lack of activity of quinoxaline-containing derivatives (**10a**, **19a**, and **19b**) could be explained by differences in their binding modes, possibly caused by the increased bulkiness of the aromatic fragment. However, the predicted poses of **10a**, **19a**, and **19b** (as exemplified by **19b** in Figure S2) were comparable to the poses of the above-mentioned derivatives **6b** and **15b**. This indicates that the lack of activity of quinoxaline derivatives is likely related only to the physico-chemical properties (decreased water solubility) as discussed above.

#### 2.5.2. Investigation of Binding Mode Stability

Thus far, none of the structurally related compounds described in Figure 1 have been cocrystallized in the FabH. The “correct” binding mode is thus unknown. We decided to test the stability of binding mode 1 using both **6b** and **15b** with short molecular dynamics simulations. This method has already been established to accurately disqualify decoy poses from the correct (crystallographic) ones [48]. The biological assembly of the MtFabH is defined as a homodimer, coordinates of both subunits in PDB ID: 1U6S are identical (RMSD after superposition to all residues is 0.3 Å, with respect to pocket residues < 0.1 Å). Therefore, simulating the chosen binding mode in both subunits at the same time could be considered as running two independent simulations of the same binding mode. For each ligand, to save computational time while gathering twice the amount of stability data, we introduced the highest scoring poses from docking to their respective subunits and simulated them simultaneously in the MtFabH dimer.

The docking poses were not modified in any way as we were interested in whether any preference for rotation of chloropyridine or oxazole would be observed in either ligand during the MD run. The system was minimized, heated to 300 K, and equilibrated. Three independent 50 ns production runs were started from the equilibrated system with reinitializing velocities. The stability of the binding mode was evaluated based on criteria as used by Liu and colleagues [48], i.e., binding mode would be unstable if the last 5 ns average ligand position was significantly different from the docked pose (RMSD > 2.0 Å calculated for the heavy atoms). To eliminate possible mistakes due to internal motion of the ligand or the active site, each replica was also analyzed visually. Results are summarized in Table 5.

The H-bond interaction with Ala306 was preserved in all stable poses. From the six replicas of **6b** (3 runs × 2 subunits), the pose was concluded as unstable only once (RMSD to the docked pose 4.19 Å). In some replicas, we found that the oxazole core rotated, reaching the position of the second docked pose. However, it did not affect the general stability of the binding mode. The rotation of the oxazole ring seemed to be influenced by the H-bond with Ser276 (Ser-OH…N_oxazole_).

Due to the increased number of degrees of freedom (rotatable bonds) of **15b** and its generally higher flexibility in the active site, the RMSD of **15b** average poses was slightly higher than in **6b**. The pose was considered unstable in two cases, although the RMSD was close to the defined cut-off (see Table 5). The oxazole interaction with Ser276 also occurred, but less frequently than in **6b,** which is expected due to the inability of the 4-phenyloxazol-2-amine fragment to freely rotate in the active site. The initial position of the chlorine atom in chloropyridine did not seem to affect the stability of the binding mode. Based on the obtained RMSD (see Table 5), binding mode 1 can be considered stable for both **6b** and **15b**, and thus represents a viable binding mode of subtype I and II compounds in MtFabH. RMSD curves of the representative runs may be seen in Figure S4 in the Supplementary Materials.

As seen in Table 1, derivatives substituted in position 3 or disubstituted in positions 3 and 5 (respective to the carboxamide linker) are more active than the others. This could be rationalized by the favorable position of the substituent inside the binding pocket, which could either point toward the hydrophobic area of the tunnel surface or it can fill the hydrophobic subpocket formed by Ile156, Phe157, Leu207, Ala246, and Asn274. In the case of 3,5-disubstituted derivatives, both positions could be occupied at the same time. Indeed, as an example, we were able to dock the 3,5-disubstituted derivative **16b** in binding mode 1 (see Figure S2).

### 2.6. Cytotoxicity Screening

Cytotoxicity of the synthesized compounds was evaluated using standard hepatic cell line HepG2 using a commercial CellTiter 96 assay. The parameter IC_50_ was determined, which allowed a quantitative comparison of the toxicity among tested compounds. The IC_50_ values are presented in Table 1. Several established antituberculars are known to carry a risk of hepatotoxic behavior [49], and the cytotoxic effect on the hepatic cell line is thus a relevant surrogate.

No difference in cytotoxicity between 2-AMT and 2-AMO derivatives was observed. The majority of compounds were non-cytotoxic, and no significant decrease in cell viability was reached at the highest concentrations used (IC_50_ >1000 µM in most cases, see Table 1). We observed solubility issues with some 2-aminothiazole derivatives. Eight compounds precipitated in the incubation medium at higher concentrations. The IC_50_ values were thus impossible to determine. Hence, derivatives **6a**, **7a** were determined only up to 250 µM; **11a**, **13a**, **14b**, **18b** up to 100 µM; **10a** up to 50 µM; and **12a** up to 25 µM. All appeared non-cytotoxic up to their highest tested concentration.

The determined IC_50_ values of some antimycobacterially active compounds (e.g., **15a**, **15b**) indicated a certain degree of cytotoxicity at higher concentrations as seen in Table 1 or Table 6. Yet, the selectivity towards Mtb, expressed as the selectivity index ( $\mathrm{SI}={\mathrm{IC}}_{50}\;(µM)/{\mathrm{MIC}}_{H37\mathrm{Ra}}\;(µM)$ ), was above 10 for majority of the most active compounds (MIC < 7.81 µg/mL, see Table 6), which can be considered a reasonable starting point for further optimizations. Alongside the most active derivatives, compounds **5b**, but also **11a**, or **11b** represent reasonably selective agents worthy of further investigation. Representative IC_50_ curves can be seen in Figure S5 in the Supplementary Materials.

Three compounds presented in this study (**1a**, **2a**, **3a**―all belonging to subtype I) and similar compounds bearing a thiazole ring have been previously evaluated for inhibitory activity against methionine aminopeptidase (MetAP), a known target for both antimicrobial and anticancer agents [24,50]. In the current research, we were mainly focused on antimicrobial activity, and our in vitro cytotoxicity screening in the HepG2 cancer cell line showed that the three mentioned compounds are non-cytotoxic (IC_50_ > 1000 µM) and hence could be discarded as potential anticancer agents. In the work of Luo and colleagues [50], the inhibitory activity of **1a** on hMetAP was shown. However, the selectivity towards bacterial MetAP (tested against *S. aureus, E. coli*) was much greater (100-fold) than towards a human homologue, implying that **1a** can be further developed as an antibacterial rather than an anticancer agent, which is consistent with our findings.

In addition, structures similar to subtype II bearing the 4-phenylaminothiazole/aminooxazole substitution reported in this study were also presented as promising Hec1/Nek2 inhibitors potentially usable in anticancer therapy [51]. Such compounds have shown low µM activities against four cancer cell lines (HeLa, K562, MB46, and MB231). Yet, inhibition was highly dependent on the substitution of the phenyl ring, in contrast to our structures being unsubstituted, explaining the absence of significant cytotoxicity in our assay.

On the other hand, derivative **13a** tested in this study was proven to have a moderate cytoprotective effect (on PC12 cells), acting as a PARP inhibitor [52]. Similarly, other compounds containing 4-phenyl-substituted aminothiazole or aminooxazole with different (hetero)aroyl fragments were also tested for their antioxidant activities [53]. This could open new scope of interest for our compounds.

## 3. Materials and Methods

### 3.1. General Chemistry

All chemicals were obtained from Sigma-Aldrich/Merck (Darmstadt, Germany) or Fluorochem (Hadfield, Derbyshire, UK) and used without further purification. Solvents were bought from Penta (Prague, Czech Republic) or Merck (Germany), Milli-Q water was prepared using a Millipore purification system (Merck-Millipore, Darmstadt, Germany). Anhydrous solvents were bought from VWR (Stříbrná Skalice, Czech Republic).

Reactions were monitored on aluminium TLC Silica 60 F254 plates (Merck, Germany) and by the TLC-MS Advion Expression CMS (Advion, Ithaca, NY, USA). Uncorrected melting points were measured on Stuart SMP30 (Bibby Scientific Limited, Staffordshire, UK) using the open capillary method.

### 3.2. Synthesis

#### 3.2.1. Representative Synthetic Procedure

##### Preparation of 4-Phenyl-Substituted 2-Aminothiazole and 2-Aminooxazole

The 4-phenylthiazol-2-amine was prepared as described previously [9]. Briefly, 1 equivalent of 2-bromoacetophenone was dissolved in ethanol, charged with 1 equivalent of thiourea, and refluxed for 1 h or until disappearance of the starting material based on TLC monitoring. Residue was evaporated to dryness, extracted to ethyl acetate and Na_2_CO_3_ solution (pH 8–9), and evaporated. The compound was recrystallized from ethanol.

The 4-phenyloxazol-2-amine was prepared as described by Azzali and colleagues [8]. A 1 equivalent of 2-bromoacetophenone was heated with 10 equivalents of urea in DMF at 120 °C for 2 h or refluxed (the same equivalents) for 16 h in acetonitrile. The reaction was processed as usual (extraction to ethyl acetate, drying, evaporation) and purified using flash chromatography on a PuriFlash 5 system (Interchim, France) using UV and ELSD detection with gradient elution of hexane–acetone.

##### Coupling

Carboxylic acid (2 mmol, 1 equivalent) in a 10 mL round-bottom flask was charged with thionyl chloride (20 mmol; 10 equivalents), catalytic DMF (1–2 drops), and heated at 50–60 °C while stirring until dissolution for at least 1 h. The mixture was evaporated to dryness, and the residue was dissolved in a small amount of dry DCM (3 mL). In a separate round-bottom flask, the chosen amine (2.2 mmol; 1.1 equivalents) was dissolved (suspended) in dry DCM, and the base (pyridine or DIPEA, 6 mmol, 3 eq.) was added. The mixture was cooled on ice, and the previously prepared acyl chloride from the first flask was added dropwise upon stirring. The resulting mixture was stirred overnight at room temperature and evaporated. The crude was extracted with ethyl acetate and Na_2_CO_3_ solution (pH 8–9), then combined organic phases were washed with saturated NaCl, dried over anhydrous Na_2_SO_4_, and concentrated in vacuo. Finally, the compound was purified using flash chromatography on PuriFlash 5 (Interchim, Montluçon, France) using UV and ELSD detection with gradient elution of hexane–acetone.

#### 3.2.2. Spectroscopic Identification and Analytical Evaluation

The ^1^H– and ^13^C–NMR spectra were recorded on a Varian VNMR S500 (Varian, Palo Alto, CA, USA) at 500 MHz for ^1^H and 126 MHz for ^13^C or Jeol JNM-ECZ600R at 600 MHz for ^1^H and 151 MHz for ^13^C. Chemical shifts referred indirectly to tetramethylsilane via the signal of the solvent (2.50 for ^1^H and 39.7 for ^13^C in DMSO-*d_6_,* 7.25 for ^1^H and 77.19 for ^13^C in CDCl_3_-*d_3_,* 2.02 for ^1^H and 29.01 for ^13^C in acetone-*d_6_*) and reported in ppm (δ). The infrared spectra were recorded with an FT-IR Nicolet 6700 spectrometer (Thermo Scientific, Waltham, MA, USA) using the attenuated total reflectance (ATR) method on a germanium crystal.

The purity of the newly synthesized compounds was measured using a Nexera^®^ UHPLC system (Shimadzu, Kyoto, Japan) coupled with a PDA detector (SPD-M20A) on the Ascentis^®^ C18 (100 × 3 mm, 3 μm, Supelco^®^) column using an acetonitrile/water mobile phase mixture in isocratic or gradient mode. Data were processed using LabSolutions software (v. 5.92, Shimadzu, Kyoto, Japan). The stock solution of each compound (0.5 mg/mL) was prepared by dissolving the appropriate amount in methanol. Working solutions were prepared by diluting the stock solutions with the acetonitrile/water mixture (1:1, *v*/*v*) to a concentration of 50 μg/mL. The PDA detector acquired spectra from 190 to 380 nm, and a wavelength of 254 nm was employed for purity evaluation.

The HRMS identification was performed using the Q-Exactive Focus (Thermo Scientific, San José, CA, USA) with HESI, and the data were processed with Xcalibur software (Thermo Scientific). Heated-electrospray ionization II interface (HESI-II) in positive ion mode was used with the following settings: spray voltage, 0.5–5 kV; S-lens RF level, +50 V; capillary temperature, 350 °C; auxiliary gas heater temperature off; sheet and auxiliary gas flow, 5 and 2 arbitrary units, respectively. Data were acquired in full-scan MS mode (FullMS) at resolution (*m*/Δ*m*) ≈ 70,000 with the accuracy of measuring ≤ 2.06 ppm with quadrupole filter mass range 90–450 *m*/*z*.

### 3.3. Log k’_w_ and Solubility Evaluation

The log k of the synthesized compounds was measured using the same instrument (single measurement per concentration), software, and column as was used in the purity evaluation. Working solutions were diluted as described above for purity determination. The log k parameter was calculated after the UHPLC measurements using isocratic mode with mobile phase acetonitrile (B)/water (A) in a set of concentrations of 40, 45, 50% and 20, 30, 40% B for subtype I and subtype II, respectively. Log k values were extrapolated to 0% of B to calculate the log k’_w_ as described previously [26] using the least squares linear regression in MS Excel for Microsoft 365 (Microsoft Corporation, Redmond, WA, USA), R^2^ for individual compounds were 0.953–0.999.

A correlation plot of log k’_w_–log *P* (Silicos-IT) was constructed using scikit-learn v.1.0.2 (https://scikit-learn.org/stable/index.html (accessed on 8 April 2022) [54]) and matplotlib 3.4.1 (https://matplotlib.org/ (accessed on 8 April 2022) [55]) in Python 3.8. The ordinary least squares linear regression method was used as defined by LinearRegression class in scikit-learn.

Water solubility was investigated using the kinetic solubility method [8]. The sample (1 mg) was dissolved in the smallest amount of DMSO needed, 25 or 50 μL for subtype I and subtype II compounds, respectively. The samples were mixed at 18 °C in a thermomixer (Thermomixer comfort, Eppendorf, Hamburg, Germany) for 1 h. After mixing, the samples were diluted with water to a concentration of 500 μM, and the precipitate was observed. The sample was filtered through a 0.22 μm filter and diluted to be evaluated by UHPLC. The log *S* parameter was calculated from the experimental solubility in molar concentration. The samples were measured using the same instrument, software, and column as used in the purity evaluation.

### 3.4. Antimicrobial and Cytotoxicity Screening

Antimicrobial activity and cytotoxicity screenings were performed as published previously [24,56]. The full description of the used methodology is available in the Supplementary Materials.

### 3.5. In Silico Studies

#### 3.5.1. Docking

Docking studies were performed using Dock utility in MOE 2020.0901 [57] (Chemical Computing Group, Montreal, QC, Canada). Mycobacterial FabH (MtFabH, PDB ID: 1U6S) was downloaded from the RCSB PDB database. Residue Ala112 was mutated back to Cys112, and the most energetically favorable sidechain rotamer was generated and minimized in MOE. Solvent molecules were removed. No energy minimization was done. Protein and cocrystallized ligand (DDC, dodecyl-CoA) were prepared as usual; hydrogens were added, missing and/or incorrect amino acids were corrected, and the protonation states (at pH 7.4) of the ligand and protein residues were adjusted using Protonate3D [58]. The system was charged using Amber14:EHT force field. Compounds for docking were drawn in ChemDraw 20.0 (PerkinElmer Informatics, Waltham, MA, USA), prepared (assignment of protonation state, partial charge definition), and subsequently minimized to RMS = 0.00001 kcal.mol^−1^Å^−1^. Rigid docking was used. The binding site for docking was defined as all residues with at least one atom within 4.5 Å of the selected cocrystallized ligand atoms (the adenosine part of dodecyl-CoA was ignored as it is outside of the binding pocket). Compounds were docked to both binding sites in subunits A and B. The ligand was placed using Triangle Matcher and scored with the London dG scoring function. The thirty best poses were refined (minimized inside the rigid receptor) and rescored using the GBVI/WSA dG scoring function. Poses were analyzed visually and based on the score.

#### 3.5.2. Molecular Dynamics

Inputs for the molecular dynamics (MD) simulations were prepared in MOE and poses from the docking were used as inputs. The same force field (Amber14:EHT) and parameters for the ligands and receptor as for the docking were used. The system was solvated using TIP3P waters in a 10 Å margin periodic boundaries box, neutralized, and buffered using NaCl (c = 0.1M). The cut-off distance was set to 10 Å. Simulations were run on GPU clusters using NAMD. The temperature was controlled by Langevin dynamics, and the pressure was treated using Nosé–Hoover–Langevin piston pressure control, both as implemented in NAMD. Long-range electrostatics were treated using the particle mesh Ewald (PME). All heavy atom–hydrogen bonds were constrained using the SHAKE algorithm and a 2fs time step was used. Restraints to heavy atoms of the protein backbone and ligand were applied (force constant defined by consref = 4, consexp = 2) in the initial stages of the MD protocol (see below).

##### MD Protocol:

1. Restrained minimization—10 ps.
2. Unrestrained minimization—10 ps.
3. Restrained NVT heating—504 ps—gradual heating 0 to 300 K (force constant reduced to 2).
4. Restrained NPT equilibration—500 ps (T = 300 K, P = 1 bar, same constraints as for heating).
5. Restrained NPT equilibration—2000 ps constraints gradually turned off (T = 300 K, P = 1 bar).
6. NPT equilibration—2000 ps (T = 300 K, P = 1 bar).
7. NPT production phase—50,000 ps (50 ns) (T = 300 K, P = 1 bar).

Minimization, heating, and equilibration were done using NAMD 2.10. For each ligand, we then ran 3 independent replicas starting from the equilibrated state with reinitialization of the velocities. Reaching the equilibrated state was checked by the root mean-square deviation (RMSD) curves calculated using MDAnalysis 2.0.0 [59,60] as presented in Figure S3 in the Supplementary Materials. The production phase was run using the newest NAMD 3 alpha 9, as it can efficiently use the computation capabilities of modern GPU accelerators.

RMSD used for binding mode stability assessment was calculated from an average pose of the ligand from the last 5 ns of the production run relative to the docked pose, superposed on the active site residues (within 4 Å of the ligand). Production run trajectories were analyzed visually in MOE and by RMSD plots calculated by MDAnalysis 2.0.0.

## 4. Conclusions

In conclusion, we identified a number of compounds containing 2-aminooxazole fragments that are highly active against mycobacterial species, including multi-drug-resistant Mtb. These were similarly or even more potent than their isosteres containing 2-aminothiazole moiety, often preferred in the design of antimicrobial agents. Based on the obtained results of lipophilicity and water solubility, we concluded that improved hydrophilicity of the oxazole title compounds is the probable reason for the higher activity when compared to thiazoles. This trend was more profound within the structurally less complex subtype I than in subtype II compounds, bearing a phenyl substitution on the 2-aminooxazolyl or thiazolyl core. Several 2-aminooxazole derivatives showed broad-spectrum activity against the screened microorganisms. Tested compounds were non-cytotoxic.

Unless extremely unfavorable physical–chemical properties are the cause of inactivity, exchanging sulfur with oxygen in antimicrobial 2-aminothiazoles on its own is thus probably not sufficient to make a drug from a non-drug. Yet, the use of 2-AMO might prove a useful strategy in smaller drugs to attain desired polarity and hydrophilicity in hit-to-lead optimizations, especially in cases when 2-AMT-containing compounds are excluded from in vitro evaluations due to the application of PAINS filters or due to insufficient water solubility in assays.

We also studied a potential target of the title compounds, MtFabH, and we identified a stable binding mode involving interactions within the described active site. Interactions involved H-bonds and π-interactions with the catalytic triad and other close-by residues. We also rationalized improved antimycobacterial activity due to the favorable 3-substitution of the aroyl fragment. Further computational analyses of binding mode 1 were beyond our intentions, but they are highly encouraged alongside crystallographic determination of the “correct” binding mode.

Considering that only a few substituents were investigated in this study, the introduction of other small to medium-sized substituents to either fragment of the title compounds, as done previously, e.g., by Meissner [21] or Zitko [11], could show more detailed SAR in the 2-aminooxazole series, which was not the main goal of this study but it is planned in future.

## Figures and Tables

**Table 1:** Structures, log k’_w_, HepG2 cytotoxicity, and MIC against Mtb H37Ra of the title compounds.

Structure	Code	Ar	X	log k’_w_	HepG2 IC_50_ (µM)	Mtb H37Ra MIC (µg/mL)
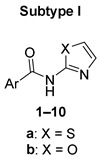	**1a**	pyridin-2-yl	S	1.857	>1000 *	31.25
	**1b**	pyridin-2-yl	O	0.854	>1000 *	62.5
	**2a**	pyridin-3-yl	S	1.251	>1000 *	250
	**2b**	pyridin-3-yl	O	0.436	>1000 *	31.25
	**3a**	pyridin-4-yl	S	1.306	>1000 *	250
	**3b**	pyridin-4-yl	O	0.396	>1000 *	15.625
	**4b**	5-Me-pyridin-3-yl	O	0.888	>1000 *	7.81
	**5b**	2-Me-pyridin-4-yl	O	0.714	>1000 *	3.91
	**6a**	2-Cl-pyridin-4-yl	S	2.013	>250 **	≥500
	**6b**	2-Cl-pyridin-4-yl	O	1.136	664.1	3.125
	**7a**	2-Cl-6-Me-pyridin-4-yl	S	2.319	>250 **	≥500
	**7b**	2-Cl-6-Me-pyridin-4-yl	O	1.430	959.4	<3.91
	**8a**	pyrazin-2-yl	S	1.222	n.d.	62.5
	**8b**	pyrazin-2-yl	O	0.154	n.d.	31.25
	**9a**	5-Cl-pyrazin-2-yl	S	1.941	n.d.	31.25
	**9b**	5-Cl-pyrazin-2-yl	O	0.958	n.d.	31.25
	**10a**	quinoxalin-2-yl	S	2.530	>50 **	≥250
	**10b**	quinoxalin-2-yl	O	1.493	>1000 *	15.625
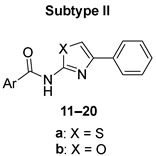	**11a**	pyridin-2-yl	S	3.102	>100 **	3.91
	**11b**	pyridin-2-yl	O	2.038	883.4	3.91
	**12a**	pyridin-3-yl	S	2.131	>25 **	≥500
	**12b**	pyridin-3-yl	O	1.118	610.3	125
	**13a**	pyridin-4-yl	S	2.190	>100 **	7.81
	**13b**	pyridin-4-yl	O	1.163	879.3	31.25
	**14b**	5-Me-pyridin-3-yl	O	1.478	>100 **	≥250
	**15a**	2-Cl-pyridin-4-yl	S	3.036	102.6	3.91
	**15b**	2-Cl-pyridin-4-yl	O	1.992	136.1	7.81
	**16a**	2-Cl-6-Me-pyridin-4-yl	S	3.314	n.d.	7.81
	**16b**	2-Cl-6-Me-pyridin-4-yl	O	2.251	n.d.	15.625
	**17a**	pyrazin-2-yl	S	2.365	n.d.	>50 [11]
	**17b**	pyrazin-2-yl	O	1.306	>1000 *	15.625
	**18a**	5-Cl-pyrazin-2-yl	S	3.173	n.d.	>100 [11]
	**18b**	5-Cl-pyrazin-2-yl	O	2.073	>100 **	15.625
	**19a**	quinoxalin-2-yl	S	3.583	n.d.	≥500
	**19b**	quinoxalin-2-yl	O	2.465	n.d.	≥500
	**20b**	phenyl	O	2.090	330.3	62.5
	**CIP**	-	-	-	-	0.25
	**INH**	-	-	-	-	0.25
	**RIF**	-	-	-	-	0.003–0.0015

* IC_50_ above the highest tested concentration; ** exact IC_50_ value could not be determined due to insolubility in the testing medium at higher concentrations; CIP—ciprofloxacin; INH—isoniazid; RIF—rifampicin; n.d.—not determined.

**Table 2:** Water solubility and log S* of the investigated compounds.

Compound	Solubility (μg/mL)	Relative Solubility ^1^	Exp. log S *	Calc. log S **
**6a**	29.93	1	−3.90	−3.38
**6b**	no precipitate	n.d.	n.d.	−2.43
**12a**	0.28	1	−6.00	−4.11
**12b**	115.65	413	−3.36	−3.16
**15a**	2.10	1	−5.18	−5.11
**15b**	123.99	59	−3.38	−4.15

^1^ Relative to the corresponding thiazole isostere; * calculated based on experimentally measured water solubility (in molar concentration); ** values determined using the algorithm of Ali and colleagues [32] calculated by SwissADME; n.d.—not determined.

**Table 3:** Antimycobacterial activity of compounds against Mtb H37Rv virulent strain and MDR clinical isolates. MIC values are in µg/mL.

Compound	MIC Mtb H37Rv	MIC Mtb IZAK	MIC Mtb MATI
**6b**	6.25	3.13	3.13
**7b**	6.25	3.13	3.13
**CIP**	0.2	0.2	0.2
**EMB**	0.39	1.56	1.56
**INH**	0.39	12.5	12.5 (>12.5)

CIP—ciprofloxacin; EMB—ethambutol; INH—isoniazid.

**Table 4:** Interactions of binding mode 1 observed in the docking studies.

Compound	Score	Ligand Atom/ Fragment	Receptor Atoms	Interaction Type	Distance (Å)	Energy (kcal/mol)
**6b**	−6.4	O (carbonyl)	NH_BB_ Cys112	HBA	3.20	−0.7
		O (carbonyl)	NH_BB_ Ala306	HBA	3.00	−1.9
		Pyridine	NH_SC_ Asn274	NH-π	3.73	−0.8
**15b**	−8.1	O (carbonyl)	NH_BB_ Cys112	HBA	3.21	−0.5
		O (carbonyl)	NH_BB_ Ala306	HBA	3.06	−1.8
		Pyridine	NH_SC_ Asn274	NH-π	3.75	−0.8

X_BB_—backbone atom; X_SC_—side chain atom. Energies were calculated using Amber14:EHT force field. Distances are presented between heavy atoms (H-bonds) or heavy atom–centroid (NH-π).

**Table 5:** Stability of binding mode 1 of both investigated ligands, expressed as RMSD (Å) ^1^ to the docking pose.

**Ligand**	**Replica**						**Result**
	1	2	3	4	5	6	
**6b**	0.86	1.56	1.32	1.69	4.19	0.79	Stable
**15b**	2.25	1.49	2.24	1.83	1.30	1.98	Stable

^1^ Average from the last 5 ns of the production run.

**Table 6:** Cytotoxicity and selectivity of the most antimycobacterially active compounds.

Compound	HepG2 IC_50_ (µM)	MIC_H37Ra_ (µg/mL)	MIC_H37Ra_ (µM)	SI
**4b**	>1000	7.81	38.4	>26.0
**5b**	>1000	3.91	19.2	>52.0
**6b**	664.1	3.125	14.0	47.5
**7b**	959.4	<3.91	<16.5	>58.3
**11a**	>100	3.91	13.9	>7.2
**11b**	>1000	3.91	14.7	>67.8
**13a**	>100	7.81	27.8	>3.6
**15a**	102.6	3.91	12.4	8.3
**15b**	136.1	7.81	26.1	5.2

## Data Availability

The data presented in this study are available within the article and Supplementary materials.

## Supplementary Materials

The following supporting information can be downloaded at: https://www.mdpi.com/1424-8247/15/5/580/s1. CSV file containing calculated and experimentally determined properties and descriptors. Supplementary material file containing description of the used methods, analytical data, representative NMR spectra, results of antimycobacterial screening against atypical mycobacteria, results of antibacterial and antifungal screening, and representative cytotoxicity IC50 curves, Figure S1: Correlation plot of log P (calculated by SILICOS-IT) and log k’w, Figure S2: Docked poses of 15b, 16b, and 19b in MtFabH, Figure S3: Backbone RMSD of the MD minimization–heating–equilibration stages of 6b-MtFabH and 15b-MtFabH complexes, Figure S4: Representative RMSD curves for the production runs of the derivatives 6b and 15b, Figure S5: Representative cytotoxicity IC50 curves of the derivatives 6b and 15b, Table S1: Susceptibility profiles of tested MDR Mtb strains, Table S2: Antimycobacterial activity against fast-growing and atypical mycobacteria, Table S3: Antibacterial activity against tested bacterial species, Table S4: Antifungal activity against tested fungal species.
