# Correction: Juhás et al. Improving Antimicrobial Activity and Physico-Chemical Properties by Isosteric Replacement of 2-Aminothiazole with 2-Aminooxazole. *Pharmaceuticals* 2022, *15*, 580

**DOI:** 10.3390/ph16030384

**Published:** 2023-03-02

**Authors:** Martin Juhás, Andrea Bachtíková, Daria Elżbieta Nawrot, Paulína Hatoková, Vinod Sukanth Kumar Pallabothula, Adéla Diepoltová, Ondřej Janďourek, Pavel Bárta, Klára Konečná, Pavla Paterová, Vít Šesták, Jan Zitko

**Affiliations:** 1 Faculty of Pharmacy in Hradec Králové, Charles University, Akademika Heyrovského 1203, 500 05 Hradec Králové, Czech Republic; 2 Department of Clinical Microbiology, University Hospital Hradec Králové, Sokolská 581, 500 05 Hradec Králové, Czech Republic; 3 Department of Clinical Biochemistry and Diagnostics, Faculty of Medicine in Hradec Králové, University Hospital, Sokolská 581, 500 05 Hradec Králové, Czech Republic

In the original publication [1], there was a mistake in assigning individual compounds to their structural subtype in Figure 2 and Table 1. In correction, subtype I comprises compounds **1**–**10**, and subtype II comprises compounds **11**–**20**. Also, for compounds **11**–**13** in Table 1, we unified the naming of the Ar substituent, using the pyridinyl convention instead of pyridyl (to achieve consistency with the rest of the paper). The corrected Figure 2 and Table 1 appear below. We made the same changes to Table S2 in the Supplementary Material. The authors apologize for any inconvenience caused and state that the scientific conclusions are unaffected. This correction was approved by the academic editor. The original publication has also been updated.

## Figures and Tables

**Figure 2:**
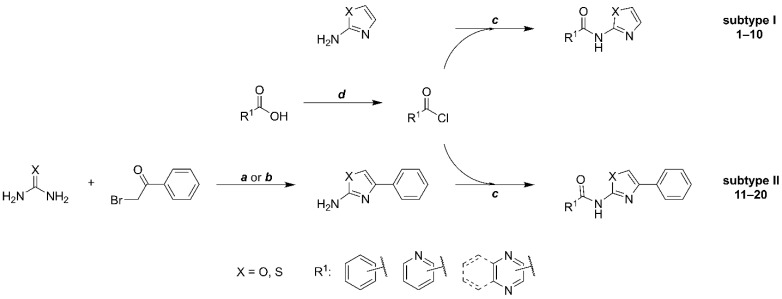
Synthetic procedure used to prepare title compounds. Conditions: **a:** (for X = S) 1.1 eq. urea, in EtOH, reflux 2 h; **b:** (for X = O) 10 eq. urea, in MeCN, reflux 16 h or in DMF, 120 °C 2 h; **c**: 1 eq. acyl chloride, 3 eq. DIPEA or pyridine, in DCM, overnight; **d**: 10 eq. thionyl chloride, catalytic DMF.

**Table 1:** Structures, log k’_w_, HepG2 cytotoxicity, and MIC against Mtb H37Ra of the title compounds.

Structure	Code	Ar	X	log k’w	HepG2 IC50 (µM)	Mtb H37Ra MIC (µg/mL)
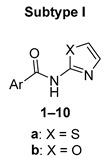	**1a**	pyridin-2-yl	S	1.857	>1000 *	31.25
	**1b**	pyridin-2-yl	O	0.854	>1000 *	62.5
	**2a**	pyridin-3-yl	S	1.251	>1000 *	250
	**2b**	pyridin-3-yl	O	0.436	>1000 *	31.25
	**3a**	pyridin-4-yl	S	1.306	>1000 *	250
	**3b**	pyridin-4-yl	O	0.396	>1000 *	15.625
	**4b**	5-Me-pyridin-3-yl	O	0.888	>1000 *	7.81
	**5b**	2-Me-pyridin-4-yl	O	0.714	>1000 *	3.91
	**6a**	2-Cl-pyridin-4-yl	S	2.013	>250 **	≥500
	**6b**	2-Cl-pyridin-4-yl	O	1.136	664.1	3.125
	7a	2-Cl-6-Me-pyridin-4-yl	S	2.319	>250 **	≥500
	**7b**	2-Cl-6-Me-pyridin-4-yl	O	1.430	959.4	<3.91
	**8a**	pyrazin-2-yl	S	1.222	n.d.	62.5
	**8b**	pyrazin-2-yl	O	0.154	n.d.	31.25
	**9a**	5-Cl-pyrazin-2-yl	S	1.941	n.d.	31.25
	**9b**	5-Cl-pyrazin-2-yl	O	0.958	n.d.	31.25
	**10a**	quinoxalin-2-yl	S	2.530	>50 **	≥250
	**10b**	quinoxalin-2-yl	O	1.493	>1000 *	15.625
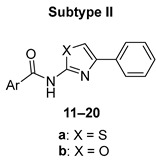	**11a**	pyridin-2-yl	S	3.102	>100 **	3.91
	**11b**	pyridin-2-yl	O	2.038	883.4	3.91
	**12a**	pyridin-3-yl	S	2.131	>25 **	≥500
	**12b**	pyridin-3-yl	O	1.118	610.3	125
	**13a**	pyridin-4-yl	S	2.190	>100 **	7.81
	**13b**	pyridin-4-yl	O	1.163	879.3	31.25
	**14b**	5-Me-pyridin-3-yl	O	1.478	>100 **	≥250
	**15a**	2-Cl-pyridin-4-yl	S	3.036	102.6	3.91
	**15b**	2-Cl-pyridin-4-yl	O	1.992	136.1	7.81
	**16a**	2-Cl-6-Me-pyridin-4-yl	S	3.314	n.d.	7.81
	**16b**	2-Cl-6-Me-pyridin-4-yl	O	2.251	n.d.	15.625
	**17a**	pyrazin-2-yl	S	2.365	n.d.	>50 [11]
	**17b**	pyrazin-2-yl	O	1.306	>1000 *	15.625
	**18a**	5-Cl-pyrazin-2-yl	S	3.173	n.d.	>100 [11]
	**18b**	5-Cl-pyrazin-2-yl	O	2.073	>100 **	15.625
	**19a**	quinoxalin-2-yl	S	3.583	n.d.	≥500
	**19b**	quinoxalin-2-yl	O	2.465	n.d.	≥500
	**20b**	phenyl	O	2.090	330.3	62.5
	**CIP**	-	-	-	-	0.25
	**INH**	-	-	-	-	0.25
	**RIF**	-	-	-	-	0.003–0.0015

* IC_50_ above the highest tested concentration; ** exact IC_50_ value could not be determined due to insolubility in the testing medium at higher concentrations; CIP—ciprofloxacin; INH—isoniazid; RIF—rifampicin; n.d.—not determined.
