# Supplementary material for: Improving Antimicrobial Activity and Physico-Chemical Properties by Isosteric Replacement of 2-Aminothiazole with 2-Aminooxazole
Source: Pharmaceuticals (Basel). 2022 May 6;15(5):580. doi: 10.3390/ph15050580 (PMC9143880; doi:10.3390/ph15050580)
Supplement: Supplementary file 1 [file pharmaceuticals-15-00580-s001.zip › pharmaceuticals-1698094-supplementary.pdf]

Improving antimicrobial activity and physico-chemical properties by isosteric replacement of 2-aminothiazole with 2-aminooxazole

Martin Juhás[1]*, Andrea Bachtíková[1], Daria Elżbieta Nawrot[1], Paulína Hatoková[1], Vinod Sukanth Kumar Pallabothula[1], Adéla Diepoltová[1], Ondřej Janďourek[1], Pavel Bárta[1], Klára Konečná[1], Pavla Paterová[2], Vít Šesták[3], Jan Zitko[1]*

[1] Faculty of Pharmacy in Hradec Králové, Charles University, Akademika Heyrovského 1203, 500 05 Hradec Králové, Czech Republic; bachtikand@faf.cuni.cz (A.B.); nawrotd@faf.cuni.cz (D.E.N); hatokovp@faf.cuni.cz (P.H.); pallabov@faf.cuni.cz (V.S.K.P.); diepolta@faf.cuni.cz (A.D); jando6aa@faf.cuni.cz (O.J.); pavel.barta@faf.cuni.cz (P.B.); konecna@faf.cuni.cz (K.K.)

[2] Department of Clinical Microbiology, University Hospital Hradec Králové, Sokolská 581, 500 05 Hradec Králové, Czech Republic; pavla.paterova@fnhk.cz (P.P.)

[3] Department of Clinical Biochemistry and Diagnostics, University Hospital and Faculty of Medicine in Hradec Králové, Sokolská 581, 500 05 Hradec Králové, Czech Republic; vit.sestak@fnhk.cz (V.Š.)

*Correspondence: juhasm@faf.cuni.cz (M.J.); jan.zitko@faf.cuni.cz (J.Z.)
Tel.: +420-495-067-272 (M.J.); +420-495-067-409 (J.Z.).

Supplementary materials

Contents

1. Materials and Methods 2
  1.1. Antimicrobial screening 2
    1.1.1. Antimycobacterial screening against Mtb H37Ra, *M. smegmatis*, *M. aurum*, *M. avium*, *M. kansasii* 2
    1.1.2. Antimycobacterial screening against Mtb H37Rv and MDR strains of Mtb 3
    1.1.3. Antibacterial screening 4
    1.1.4. Antifungal screening 5
  1.2. Cytotoxicity screening 6
2. Results 7
  2.1. Analytical description of compounds 7
    2.1.1. Representative NMR spectra 13
  2.2. Investigation of lipophilicity 15
  2.3. *In silico* studies 15
    2.3.1. Docking 15
    2.3.2. Investigation of binding mode stability 16
  2.4. In vitro screening of antimycobacterial activity 18
  2.5. In vitro screening of antibacterial activity 19
  2.6. In vitro screening of antifungal activity 20
  2.7. Cytotoxicity screening 21
3. Description of the supplied csv file 22
References 22

1. Materials and Methods

1.1. Antimicrobial screening

1.1.1. Antimycobacterial screening against Mtb H37Ra, *M. smegmatis*, *M. aurum*, *M. avium*, *M. kansasii*

The antimycobacterial assay was performed with fast growing *Mycolicibacterium smegmatis* DSM 43465 (ATCC 607), *Mycolicibacterium aurum* DSM 43999 (ATCC 23366), and non-tuberculous (atypical) mycobacteria, namely *Mycobacterium avium* DSM 44156 (ATCC 25291), *Mycobacterium kansasii* DSM 44162 (ATCC 12478) from German Collection of Microorganisms and Cell Cultures (Braunschweig, Germany), and with an avirulent strain of *Mycobacterium tuberculosis* H37Ra ITM-M006710 (ATCC 9431) from Belgian Co-ordinated Collections of Micro-organisms (Antwerp, Belgium). The technique used for activity determination was the microdilution broth panel method using 96-well microtitration plates[1]. The culture medium was Middlebrook 7H9 broth (Merck, Darmstadt, Germany) enriched with 0.4% glycerol (Merck, Darmstadt, Germany) and 10% Middlebrook OADC growth supplement (Himedia, Mumbai, India).

The mycobacterial strains were cultured on supplemented Middlebrook 7H9 agar, and suspensions were prepared in supplemented Middlebrook 7H9 broth. The final density was adjusted to value 1.0 according to the McFarland scale and diluted in the ratio 1:20 (for fast-growing mycobacteria) or 1:10 (for the rest of mycobacteria) with broth.

The tested compounds were dissolved in DMSO (Sigma-Aldrich), then Middlebrook 7H9 broth was added to obtain a concentration of 2000 µg/mL. The standards used for activity determination were isoniazid (INH), rifampicin (RIF), and ciprofloxacin (CIP) (Merck). The final concentrations were reached by binary dilution and the addition of mycobacterial suspension and were set as 500, 250, 125, 62.5, 31.25, 15.625, 7.81, and 3.91 µg/mL. The final concentration of DMSO in any well did not exceed 2.5% (*v*/*v*) and did not affect the growth of mycobacteria. Positive (broth, DMSO, bacteria) and negative (broth, DMSO) growth controls were included.

The plates were sealed with polyester adhesive film and incubated in the dark at 37 °C without agitation. The addition of 0.01% solution of resazurin sodium salt followed after 48 h of incubation for *M. smegmatis*, after 72 h of incubation for *M. aurum,* after 96 h of incubation for *M. avium* and *M. kansasii*, and after 120 h of incubation for Mtb H37Ra, respectively. The microtitration panels were then incubated for a further 2.5 h for the determination of activity against *M. smegmatis*, 4 h for *M. aurum*, 5-6 h for *M. avium* and *M. kansasii*, and 18 h for Mtb H37Ra, respectively. The antimycobacterial activity was expressed as the minimum inhibitory concentration (MIC). The MIC (in µg/mL) was determined on the basis of stain color change (blue color—active; pink color—not active). All experiments were conducted in duplicates.

1.1.2. Antimycobacterial screening against Mtb H37Rv and MDR strains of Mtb

Microdilution method based on Microplate Alamar Blue Assay (MABA) was applied[1]. Tested strain Mtb H37Rv CNCTC My 331/88 (ATCC 27294) was obtained from the Czech National Collection of Type Cultures (CNCTC), National Institute of Public Health (Prague, Czech Republic). Multi-drug resistant strains of Mtb laboratory ID designation IZAK and MATI were obtained from the Department of Clinical Microbiology, University Hospital Hradec Králové from Dr. Pavla Paterová. Middlebrook 7H9 broth of declared pH 6.6 (Sigma-Aldrich) enriched with 0.4% of glycerol (Sigma-Aldrich) and 10% of OADC growth supplement (Himedia, Mumbai, India) was used for cultivation.

Tested compounds were dissolved and diluted in DMSO and mixed with broth (25 µL of DMSO solution in 2.475 mL of broth) and placed (100 µL) into microplate wells. Mycobacterial inocula were suspended in isotonic saline solution and the density was adjusted to 0.5–1.0 according to McFarland scale. These suspensions were diluted by 10^{-1} and used to inoculate the testing wells, adding 100 µL of mycobacterial suspension per well. Final concentrations of tested compounds in wells were 100, 50, 25, 12.5, 6.25, 3.13 and 1.56 µg/mL. INH was used as standard (inhibition of growth). Positive control (visible growth) consisted of broth plus mycobacterial suspension plus DMSO. A total of 30 µL of Alamar Blue working solution (1:1 mixture of 0.02% resazurin sodium salt (aq. sol.) and 10% Tween 80) was added after five days of incubation. Results were then determined after 24 h of incubation. The MIC (in µg/mL) was determined as the lowest concentration that prevented the blue to pink color change. All experiments were conducted in duplicates.

Susceptibility profiles for the used MDR Mtb strains

Mtb laboratory ID IZAK, isolated from 63-years-old man from bronchial aspirate in 2020, tested and interpreted according to CLSI (Clinical and Laboratory Standards Institute) breakpoints in 2020.
Mtb laboratory ID MATI, isolated from 23-years-old man from sputum in 2021, tested and interpreted according to CLSI breakpoints in 2021. Drug susceptibility is summarized in **Table S1**.

Table S1. *Susceptibility profiles of tested MDR Mtb strains.*

Laboratory ID	Drug	Concentration (µg/mL)	Susceptibility
IZAK	STM	4	resistant
	INH	4	resistant
	RIF	>8	resistant
	EMB	**0.5**	**sensitive**
	PZA	>16	resistant
MATI	STM	>16	resistant
	INH	>8	resistant
	RIF	>8	resistant
	EMB	**0.5**	**sensitive**
	PZA	>128	resistant

STM – streptomycin; INH – isoniazid; RIF – rifampicin; EMB – ethambutol; PZA – pyrazinamide

1.1.3. Antibacterial screening

The microdilution broth method was performed according to EUCAST (The European Committee on Antimicrobial Susceptibility Testing) instructions[2] with slight modifications. Eight tested bacterial strains (four G+ and four G-) were purchased from the Czech Collection of Microorganisms (CCM, Brno, Czech Republic) or from the German Collection of Microorganisms and Cell Cultures (DSM, Braunschweig, Germany): *Staphylococcus aureus* subsp. *aureus* CCM 4223 (ATCC 29213), methicillin-resistant *Staphylococcus aureus* subsp. *aureus* (MRSA) CCM 4750 (ATCC 43300), *Staphylococcus epidermidis* CCM 4418 (ATCC 12228), *Enterococcus faecalis* CCM 4224 (ATCC 29212), *Escherichia coli* CCM 3954 (ATCC 25922), *Klebsiella pneumoniae* CCM 4415 (ATCC 10031), *Acinetobacter baumannii* DSM 30007 (ATCC 19606), *Pseudomonas aeruginosa* CCM 3955 (ATCC 27853). The cultivation was done in Cation-adjusted Mueller-Hinton broth (CAMHB, M-H 2 Broth, Merck, Darmstadt, Germany) at 35 ± 2 °C.

Tested compounds were dissolved in DMSO (Merck) to produce stock solutions. The final concentration of DMSO in the cultivation medium did not exceed 1% (v/v) of the total solution composition and did not affect the growth of bacteria. Positive growth controls consisted of test microbe solely, while negative growth controls consisted of cultivation medium and DMSO. Antibacterial activity was expressed as minimum inhibitory concentration (MIC, in µM) after 24 and 48 h of static incubation in the dark and humidified atmosphere, at 35 ± 2 °C. Visual inspection and metabolic activity indicator, Alamar Blue (AlamarBlue™ Cell Viability reagent, ThermoFisher Scientific, USA), were used for MIC endpoint evaluation. The internal quality standards of gentamicin and ciprofloxacin (both from Merck) were involved in assays (for MIC of standards, see below). All experiments were conducted in duplicates.

Results of internal quality controls (standards) in antibacterial screening

**Internal quality control**	**ciprofloxacin (µg/mL)**		**gentamicin (µg/mL)**	
**Bacterial strain**	**MIC (spectrophotometric detection*)**	**MIC (visual detection**)**	**MIC (spectrophotometric detection*)**	**MIC (visual detection**)**
Staphylococcus aureus subsp. *aureus* CCM 4223 (ATCC 29213)	0.256	0.256	1	0.5
Staphylococcus aureus subsp. *aureus* (MRSA) CCM 4750 (ATCC 43300)	0.128	0.128	> 8	> 8
Staphylococcus epidermidis CCM 4418 (ATCC 12228)	0.256	0.128	0.0625	0.0625
Enterococcus faecalis CCM 4224 (ATCC 29212)	1.024	1.024	> 8	> 8
Escherichia coli CCM 3954 (ATCC 25922)	0.008	0.008	1	1
Klebsiella pneumoniae CCM 4415 (ATCC 10031)	0.064	0.128	0.5	0.5
Acinetobacter baumannii DSM 30007 (ATCC 19606)	0.512	0.512	8	8
Pseudomonas aeruginosa CCM 3955 (ATCC 27853)	0.512	0.256	0.5	0.5

Notes: Spectrophotometric detection - results were read with a microplate reader (Synergy™ HTX, BioTek Instruments, Inc., USA) at wavelength 530 nm, MIC – minimum inhibitory concentration

*The MIC of antibacterial agents is the lowest concentration giving rise to an inhibition of growth of 95% of that of the drug-free control. Results were read 24 h after incubation without agitation at 35 ± 2 °C in a humidified atmosphere.

**The MIC was determined by the naked eye in the well with the lowest drug concentration, where no visible growth of microbial agent was detected. Results were read after 24 h incubation without agitation at 35 ± 2 °C in a humidified atmosphere.

1.1.4. Antifungal screening

Antifungal activity evaluation was performed using a microdilution broth method according to EUCAST instructions[3,4] with slight modifications. Eight fungal strains (four yeast and four moulds) were used for antifungal activity screening, namely: *Candida albicans* CCM 8320 (ATCC 24433), *Candida krusei* CCM 8271 (ATCC 6258), *Candida parapsilosis* CCM 8260 (ATCC 22019), *Candida tropicalis* CCM 8264 (ATCC 750), *Aspergillus fumigatus* ATCC 204305, *Aspergillus flavus* CCM 8363, *Lichtheimia corymbifera* CCM 8077, and *Trichophyton interdigitale* CCM 8377 (ATCC 9533). Tested strains were purchased from the Czech Collection of Microorganisms (CCM, Brno, Czech Republic) or from the American Type Collection Cultures (ATCC, Manassas, VA, USA).
Tested compounds were dissolved in DMSO and diluted in a two-fold manner with RPMI 1640 medium, glutamine and 2% glucose, buffered to pH 7.0 with MOPS (3-morpholinopropane-1-sulfonic acid). The final concentration of DMSO in the testing medium did not exceed 1% (v/v) of the total solution composition. Static incubation was performed in the dark and in a humid atmosphere, at 35 ± 2 °C, for 24 and 48 h (72 and 120 h for *Trichophyton interdigitale,* respectively). Positive growth controls consisted of test microbe solely, while negative growth controls consisted of cultivation medium and DMSO. Visual inspection and metabolic activity indicator, Alamar Blue (ThermoFisher Scientific, USA), were used for MIC endpoint evaluation. The internal quality standards, amphotericin B (Merck) and voriconazole (Toronto Research Chemicals, CA) were involved in assays (for IC_{50}, IC_{90}, MIC of standards, see below). All experiments were conducted in duplicates.

Results of internal quality controls (standards) in antifungal screening

**Internal quality control**	**amphotericin B (µg/mL)**		**voriconazole (µg/mL)**	
**Yeast/mould strain**	**IC_{90} (spectrophotometric detection*)**	**MIC (visual detection**)**	**IC_{50} (spectrophotometric detection***)**	**MIC (visual detection**)**
Candida albicans CCM 8320 (ATCC 24433)	1	1	0.03	> 16
Candida krusei CCM 8271 (ATCC 6258)	1	1	0.25	> 16
Candida parapsilosis CCM 8260 (ATCC 22019)	0.5	0.5	0.03	> 16
Candida tropicalis CCM 8264 (ATCC 750)	1	1	0.0625	> 16
Aspergillus fumigatus (ATCC 204305)	4	4	0.25	1
Aspergillus flavus CCM 8363	4	4	1	> 16
Lichtheimia corymbifera CCM 8077	1	1	8	> 16
Trichophyton interdigitale CCM 8377 (ATCC 9533)	1	1	1	> 16

Notes: Spectrophotometric detection results were read with a microdilution plate reader (Synergy™ HTX) at wavelength 530 nm.
*The IC_{90} of amphotericin B is determined as the lowest concentration giving rise to an inhibition of growth of 90% of that of the drug-free control. Results were read after 24 h (yeasts) or 48 h (moulds) without agitation at 35 ± 2 °C in a humidified atmosphere.
**The MIC was determined by the naked eye in the well with the lowest drug concentration, where no visible growth of microbial agent was detected. Results were read after 24 h (yeasts) or 48 h (moulds) of incubation without agitation at 35 ± 2 °C in a humidified atmosphere.

***The IC_{50} of voriconazole is determined as the lowest drug concentration giving inhibition of growth of 50% of that of the drug-free control. Results were read after 24 h (yeasts) or 48 h (moulds) microdilution plates cultivation without agitation at t 35 ± 2 °C in a humidified atmosphere.

1.2. Cytotoxicity screening

The human hepatocellular liver carcinoma cell line HepG2 purchased from Health Protection Agency Culture Collections (ECACC, Salisbury, UK) was cultured in DMEM (Dulbecco´s Modified Eagle´s Medium – high glucose) (Sigma-Aldrich, USA) supplemented with 10% fetal bovine serum (PAA Laboratories GmbH, Pasching, Austria), 1% L-glutamine solution (Sigma-Aldrich), and non-essential amino acid solution (Sigma-Aldrich) in a humidified atmosphere containing 5% CO_2 at 37 °C. For subculturing, the cells were harvested after trypsin/EDTA (Sigma-Aldrich) treatment at 37 °C. For cytotoxicity evaluation, the cells treated with the tested substances were used as experimental groups. Untreated HepG2 cells served as controls.

The cells were seeded in a density of 10,000 cells per well in a 96-well plate 24 h prior the experiment. The next day, the cells were treated with each of the tested substances dissolved in DMSO. The tested substances were prepared at different incubation concentrations (1–1000 µM) in triplicates according to their solubility. Concurrently, the controls representing 100% cell viability, 0% cell viability (the cells treated with 10% DMSO), no cell control, and vehiculum controls were prepared in triplicates. After 24 h of incubation in a humidified atmosphere containing 5% CO_2 at 37 °C, the reagent from the kit CellTiter 96 AQueous One Solution Cell Proliferation Assay (CellTiter 96, PROMEGA, Fitchburg, WI, USA) was added. After 2 h of incubation at 37 °C, the absorbance of the samples was recorded at 490 nm (TECAN, Infinita M200, Austria). A standard toxicological parameter IC_{50} was calculated by nonlinear regression from a semilogarithmic plot of incubation concentration versus the percentage of absorbance (log(inhibitor) vs. normalized response model, least squares fit) relative to untreated controls using GraphPad Prism 9 software, (GraphPad Software, San Diego, CA, USA).

2. Results

2.1. Analytical description of compounds

***N*-(thiazol-2-yl)picolinamide (1a)**

CAS Registry Number 301208-58-2. Light yellow solid. Yield 34%. mp = 143–145°C. ^{1}H NMR (500 MHz, DMSO) δ 12.00 (s, 1H), 8.76 (ddd, *J* = 4.7, 1.7, 1.1 Hz, 1H), 8.18 (dt, *J* = 7.7, 1.1 Hz, 1H), 8.10 (td, *J* = 7.7, 1.7 Hz, 1H), 7.71 (ddd, *J* = 7.7, 4.7, 1.1 Hz, 1H), 7.58 (d, *J* = 3.6 Hz, 1H), 7.35 (d, *J* = 3.6 Hz, 1H). ^{13}C NMR (126 MHz, DMSO) δ 162.76, 157.53, 149.16, 148.15, 138.47, 138.32, 127.90, 123.13, 114.64. IR (ATR-Ge, cm^{-1}) 3182, 1675 (υNHCO), 1524, 1489, 1472, 1451, 1417. HPLC purity 99.9%. HRMS (HESI+) m/z =206.0381 (theoretical for M+H^{+} m/z = 206.0383, error –0.74 ppm).

***N*-(oxazol-2-yl)picolinamide (1b)**

Light brown solid. Yield 59%. mp = 129–131°C. ^{1}H NMR (500 MHz, DMSO) δ 11.41 (s, 1H), 8.74 (ddd, *J* = 4.7, 1.7, 1.1 Hz, 1H), 8.13 (dt, *J* = 7.7, 1.1 Hz, 1H), 8.08 (td, *J* = 7.7, 1.7 Hz, 1H), 7.99 (d, *J* = 1.1 Hz, 1H), 7.71 (ddd, *J* = 7.7, 4.7, 1.1 Hz, 1H), 7.20 (d, *J* = 1.1 Hz, 1H). ^{13}C NMR (126 MHz, DMSO) δ 162.93, 152.54, 148.75, 148.44, 138.20, 137.29, 127.63, 127.13, 122.84. IR (ATR-Ge, cm^{-1}) 3136, 1696 (υNHCO), 1585, 1476, 1459, 1429. HPLC purity 99.7%. HRMS (HESI+) m/z =190.0608 (theoretical for M+H^{+} m/z = 190.0611, error –1.51 ppm).

***N*-(thiazol-2-yl)nicotinamide (2a)**

CAS Registry Number 14397-12-7. Light brown solid. Yield 66%. mp = 195–197°C [lit. 143°C[5], 210–211°C in H_2O[6], 212°C in H_2O and pyridine[7], 213°C[8]]. ^{1}H NMR (500 MHz, DMSO) δ 12.88 (s, 1H), 9.20 (d, *J* = 2.1 Hz, 1H), 8.78 (dd, *J* = 4.8, 1.7 Hz, 1H), 8.41 (dt, *J* = 8.0, 2.1 Hz, 1H), 7.64 – 7.53 (m, 2H), 7.31 (d, *J* = 3.6 Hz, 1H). ^{13}C NMR (126 MHz, DMSO) δ 164.21, 158.97, 153.01, 149.35, 137.47, 136.05, 128.45, 123.76, 114.24. IR (ATR-Ge, cm^{-1}) 3151, 2929, 1661 (υNHCO), 1599, 1579, 1565, 1472, 1436. HPLC purity 96.0%. HRMS (HESI+) m/z = 206.0380 (theoretical for M+H^{+} m/z = 206.0383, error –1.03 ppm).

***N*-(oxazol-2-yl)nicotinamide (2b)**

CAS Registry Number 14397-13-8. Light brown solid. Yield 10%. mp = 155–160°C. ^{1}H NMR (500 MHz, DMSO) δ 11.88 (s, 1H), 9.12 (d, *J* = 2.1 Hz, 1H), 8.77 (dd, *J* = 4.8, 1.7 Hz, 1H), 8.32 (dt, *J* = 7.9, 2.1 Hz, 1H), 7.94 (d, *J* = 1.0 Hz, 1H), 7.56 (dd, *J* = 7.9, 4.8 Hz, 1H), 7.22 (d, *J* = 1.0 Hz, 1H). ^{13}C NMR (126 MHz, DMSO) δ 152.73, 149.16, 135.87, 123.55. IR (ATR-Ge, cm^{-1}) 3151, 3110, 1706 (υNHCO), 1625, 1591, 1498, 1417. HPLC purity 99.7%. HRMS (HESI+) m/z = 190.0609 (theoretical for M+H^{+} m/z = 190.0611, error –0.88 ppm).

***N*-(thiazol-2-yl)isonicotinamide (3a)**

Light brown solid. Yield 75%. mp = 194–195°C [lit. 196°C[8], lit. 196–198°C in EtOH[9]]. ^{1}H NMR (600 MHz, DMSO) δ 12.93 (s, 1H), 8.79 – 8.73 (m, 2H), 7.96 – 7.92 (m, 2H), 7.56 (d, *J* = 3.6 Hz, 1H), 7.29 (d, *J* = 3.6 Hz, 1H). ^{13}C NMR (151 MHz, DMSO) δ 164.64, 159.35, 150.94, 140.09, 137.72, 122.33, 114.77. IR (ATR-Ge, cm^{-1}) 3186, 2952, 1683 (υNHCO), 1549, 1490, 1435, 1414. HPLC purity 96.8%. HRMS (HESI+) m/z = 206.0381 (theoretical for M+H^{+} m/z = 206.0383, error –0.69 ppm).

***N*-(oxazol-2-yl)isonicotinamide (3b)**

Yellow solid. Yield 48%. mp = 175–177°C. ^{1}H NMR (500 MHz, DMSO) δ 12.03 (s, 1H), 8.82 – 8.71 (m, 2H), 7.95 (d, *J* = 1.0 Hz, 1H), 7.91 – 7.85 (m, 2H), 7.24 (d, *J* = 1.0 Hz, 1H). ^{13}C NMR (126 MHz, DMSO) δ 150.36, 140.73, 136.09, 121.83. IR (ATR-Ge, cm^{-1}) 3158, 3127, 2933, 1618 (υNHCO), 1591, 1570, 1537.

HPLC purity 99.9%. HRMS (HESI+) m/z = 190.0609 (theoretical for M+H$^+$ m/z = 190.0611, error –1.24 ppm).

5-methyl-*N*-(oxazol-2-yl)nicotinamide (4b)
Light yellow solid. Yield 11%. mp = 171–175°C. ^{1}H NMR (600 MHz, DMSO) δ 11.73 (s, 1H), 8.89 (d, *J* = 2.1 Hz, 1H), 8.58 (d, *J* = 1.0 Hz, 1H), 8.19 – 8.04 (m, 1H), 7.90 (s, 1H), 7.17 (d, *J* = 1.0 Hz, 1H), 2.34 (s, 3H). ^{13}C NMR (151 MHz, DMSO) δ 153.63, 146.91, 133.51, 129.27, 18.26. IR (ATR-Ge, cm^{-1}) 3157, 3136, 2929, 1715 (*υ*NHCO), 1625, 1436, 1416, 1301. HPLC purity 99.9%. HRMS (HESI+) m/z = 204.0765 (theoretical for M+H$^+$ m/z = 204.0767, error –1.01 ppm).

2-methyl-*N*-(oxazol-2-yl)isonicotinamide (5b)
Brown solid. Yield 71%. mp = 149–152°C. ^{1}H NMR (600 MHz, DMSO) δ 11.79 (s, 1H), 8.63 (d, *J* = 5.2 Hz, 1H), 7.95 (s, 1H), 7.75 (d, *J* = 1.0 Hz, 1H), 7.66 (d, *J* = 5.2 Hz, 1H), 7.22 (d, *J* = 1.0 Hz, 1H), 2.56 (s, 3H). ^{13}C NMR (151 MHz, DMSO) δ 158.90, 149.68, 121.14, 118.89, 24.06. IR (ATR-Ge, cm^{-1}) 3157, 3128, 1716 (*υ*NHCO), 1622, 1602, 1423, 1410, 1298. HPLC purity 99.9%. HRMS (HESI+) m/z = 204.0765 (theoretical for M+H$^+$ m/z = 204.0767, error –1.06 ppm).

2-chloro-*N*-(thiazol-2-yl)isonicotinamide (6a)
CAS Registry Number 1019457-08-9. Light brown solid. Yield 28%. mp = 152–154°C. ^{1}H NMR (500 MHz, DMSO) δ 13.06 (s, 1H), 8.62 (s, 1H), 8.10 (s, 1H), 7.96 (d, *J* = 3.6 Hz, 1H), 7.59 (s, 1H), 7.33 (d, *J* = 3.6 Hz, 1H). ^{13}C NMR (126 MHz, DMSO) δ 163.60, 159.89, 151.38, 151.27, 143.91, 136.87, 123.15, 121.89, 114.71. IR (ATR-Ge, cm^{-1}) 3077, 1592 (*υ*NHCO), 1527, 1477, 1460, 1423. HPLC purity 99.9%. HRMS (HESI+) m/z = 239.9990 (theoretical for M+H$^+$ m/z = 239.9993, error –1.33 ppm).

2-chloro-*N*-(oxazol-2-yl)isonicotinamide (6b)
Light brown solid. Yield 25%. mp = 158–162°C. ^{1}H NMR (600 MHz, DMSO) δ 12.24 (s, 1H), 8.60 (d, *J* = 5.0 Hz, 1H), 7.97 (d, *J* = 1.3 Hz, 1H), 7.92 (d, *J* = 1.0 Hz, 1H), 7.89 (dd, *J* = 5.0, 1.3 Hz, 1H), 7.27 (d, *J* = 1.0 Hz, 1H). ^{13}C NMR (151 MHz, DMSO) δ 150.89, 150.77, 122.68, 121.51. IR (ATR-Ge, cm^{-1}) 3205, 1615 (*υ*NHCO), 1591, 1576, 1541, 1463. HPLC purity 99.4%. HRMS (HESI+) m/z = 224.0217 (theoretical for M+H$^+$ m/z = 224.0221, error –1.71 ppm).

2-chloro-6-methyl-*N*-(thiazol-2-yl)isonicotinamide (7a)
CAS Registry Number 1565518-90-2. Yellow solid. Yield 26%. mp = 198.6°C. ^{1}H NMR (600 MHz, DMSO) δ 12.93 (s, 1H), 7.85 (s, 1H), 7.81 (s, 1H), 7.55 (d, *J* = 3.6 Hz, 1H), 7.29 (d, *J* = 3.6 Hz, 1H), 2.51 (s, 3H). ^{13}C NMR (151 MHz, DMSO) δ 163.73, 160.74, 150.49, 144.06, 121.34, 120.09, 114.78, 24.21. IR (ATR-Ge, cm^{-1}) 3072, 2925, 1673 (*υ*NHCO), 1543, 1489, 1386, 1325. HPLC purity 99.6%. HRMS (HESI+) m/z = 254.0144 (theoretical for M+H$^+$ m/z = 254.0149, error –1.85 ppm).

2-chloro-6-methyl-*N*-(oxazol-2-yl)isonicotinamide (7b)
Light yellow solid. Yield 13%. mp = 149–153°C. ^{1}H NMR (600 MHz, DMSO) δ 12.11 (s, 1H), 7.92 (d, *J* = 1.0 Hz, 1H), 7.76 (s, 2H), 7.25 (d, *J* = 1.0 Hz, 1H), 2.54 (s, 3H). ^{13}C NMR (151 MHz, DMSO) δ 160.18, 149.88, 135.58, 120.85, 119.58, 23.62. IR (ATR-Ge, cm^{-1}) 3263, 3107, 2926, 1593 (*υ*NHCO), 1544, 1400, 1356. HPLC purity 99.1%. HRMS (HESI+) m/z = 238.0374 (theoretical for M+H$^+$ m/z = 238.0378, error –1.74 ppm).

***N*-(thiazol-2-yl)pyrazine-2-carboxamide (8a)**
CAS Registry Number 2248961-45-9. Light brown solid. Yield 87%. mp = 170–173°C. ^{1}H NMR (600 MHz, DMSO) δ 12.45 (s, 1H), 9.28 (d, *J* = 1.5 Hz, 1H), 8.91 (d, *J* = 2.5 Hz, 1H), 8.80 (dd, *J* = 2.5, 1.5 Hz, 1H),

7.56 (d, *J* = 3.6 Hz, 1H), 7.34 (d, *J* = 3.6 Hz, 1H). ^{13}C NMR (151 MHz, DMSO) δ 162.70, 158.02, 148.76, 144.89, 144.43, 144.31, 138.52, 115.19. IR (ATR-Ge, cm^{-1}) 3067, 1667 (*υ*NHCO), 1578, 1521, 1487, 1470. HPLC purity 99.6%. HRMS (HESI+) m/z = 207.0333 (theoretical for M+H$^+$ m/z = 207.0335, error –0.93 ppm).

***N*-(oxazol-2-yl)pyrazine-2-carboxamide (8b)**

CAS Registry Number 2248961-45-8. Light brown solid. Yield 34%. mp = 160–164°C. ^{1}H NMR (600 MHz, DMSO) δ 11.64 (s, 1H), 9.23 (d, *J* = 1.5 Hz, 1H), 8.91 (d, *J* = 2.5 Hz, 1H), 8.77 (dd, *J* = 2.5, 1.5 Hz, 1H), 7.97 (d, *J* = 1.0 Hz, 1H), 7.19 (d, *J* = 1.0 Hz, 1H). ^{13}C NMR (151 MHz, DMSO) δ 162.83, 152.84, 148.86, 144.84, 144.43, 144.13, 138.01, 127.75. IR (ATR-Ge, cm^{-1}) 3159, 3126, 1694 (*υ*NHCO), 1607, 1464, 1412. HPLC purity 99.9%. HRMS (HESI+) m/z = 191.0562 (theoretical for M+H$^+$ m/z = 191.0563, error –0.66 ppm).

5-chloro-*N*-(thiazol-2-yl)pyrazine-2-carboxamide (9a)

White solid. Yield 11%. mp = 197–200°C. ^{1}H NMR (500 MHz, DMSO) δ 12.63 (s, 1H), 9.14 (d, *J* = 1.3 Hz, 1H), 8.96 (d, *J* = 1.3 Hz, 1H), 7.59 (d, *J* = 3.6 Hz, 1H), 7.37 (d, *J* = 3.6 Hz, 1H). ^{13}C NMR (126 MHz, DMSO) δ 161.74, 157.87, 151.52, 144.55, 143.75, 142.97, 137.99, 114.89. IR (ATR-Ge, cm^{-1}) 3149, 2929, 1671 (*υ*NHCO), 1512, 1482, 1463, 1424. HPLC purity 99.1%. HRMS (HESI+) m/z = 240.9940 (theoretical for M+H$^+$ m/z = 240.9945, error –2.03 ppm).

5-chloro-*N*-(oxazol-2-yl)pyrazine-2-carboxamide (9b)

Yellow solid. Yield 58%. mp = 160–164°C. ^{1}H NMR (600 MHz, DMSO) δ 11.76 (s, 1H), 9.06 (d, *J* = 1.3 Hz, 1H), 8.90 (d, *J* = 1.3 Hz, 1H), 7.96 (d, *J* = 1.0 Hz, 1H), 7.19 (d, *J* = 1.0 Hz, 1H). ^{13}C NMR (151 MHz, DMSO) δ 151.97, 144.87, 143.91, 137.83, 127.40. IR (ATR-Ge, cm^{-1}) 3142, 1705 (*υ*NHCO), 1585, 1524, 1470, 1443. HPLC purity 99.9%. HRMS (HESI+) m/z = 225.0170 (theoretical for M+H$^+$ m/z = 225.0174, error –1.52 ppm).

***N*-(thiazol-2-yl)quinoxaline-2-carboxamide (10a)**

CAS Registry Number 871561-02-3. Brown solid. Yield 22%. mp = 251.4°C. ^{1}H NMR (600 MHz, DMSO) δ 12.57 (s, 1H), 9.51 (s, 1H), 8.24 (d, *J* = 7.8 Hz, 1H), 8.18 (d, *J* = 7.8 Hz, 1H), 8.02 – 7.94 (m, 2H), 7.59 (d, *J* = 3.6 Hz, 1H), 7.36 (d, *J* = 3.6 Hz, 1H). ^{13}C NMR (151 MHz, DMSO) δ 162.85, 158.21, 144.43, 143.83, 143.66, 140.40, 138.47, 133.06, 132.03, 130.32, 129.62, 115.18. IR (ATR-Ge, cm−1) 2941, 1663 (*υ*NHCO), 1538, 1490. HPLC purity 99.8%. HRMS (HESI+) m/z = 257.0488 (theoretical for M+H$^+$ m/z = 257.0492, error –1.52 ppm).

***N*-(oxazol-2-yl)quinoxaline-2-carboxamide (10b)**

Yellow solid. Yield 19%. mp = 163–165°C. ^{1}H NMR (500 MHz, DMSO) δ 11.86 (s, 1H), 9.50 (s, 1H), 8.29 – 8.24 (m, 1H), 8.24 – 8.20 (m, 1H), 8.06 – 7.99 (m, 3H), 7.26 (d, *J* = 1.0 Hz, 1H). ^{13}C NMR (126 MHz, DMSO) δ 162.37, 152.43, 143.90, 143.39, 143.13, 139.72, 137.37, 132.50, 131.51, 129.68, 129.11, 127.21. IR (ATR-Ge, cm^{-1}) 3161, 2927, 1688 (*υ*NHCO), 1623, 1482, 1415. HPLC purity 99.6%. HRMS (HESI+) m/z = 241.0715 (theoretical for M+H$^+$ m/z = 241.0720, error –2.06 ppm).

***N*-(4-phenylthiazol-2-yl)picolinamide (11a)**

CAS Registry Number 457940-30-6. Light yellow solid. Yield 8%. mp = 128–130°C. 1H (600 MHz, $CDCl_3$) δ 11.23 (s, 1H), 8.66 (dd, J = 4.7, 1.7 Hz, 1H), 8.29 (dt, J = 7.7, 1.1 Hz, 1H), 7.93 (td, J = 7.7, 1.7 Hz, 1H), 7.90 – 7.85 (m, 2H), 7.53 (dd, J = 7.7, 4.7 1H), 7.45 – 7.39 (m, 2H), 7.36 – 7.29 (m, 1H), 7.21 (s, 1H).
13C NMR (151 MHz, $CDCl_3$) δ 162.19, 157.31, 150.63, 148.72, 147.90, 137.87, 134.53, 128.83, 128.12, 127.43, 126.21, 122.95, 108.05. IR (ATR-Ge, cm^{-1}) 3347, 3116, 2925, 1684 (*υ*NHCO), 1618, 1604, 1569,

1471, 1458. HPLC purity 99.9%. HRMS (HESI+) m/z = 282.0693 (theoretical for $M+H^+$ m/z = 282.0696, error -0.86 ppm).

***N*-(4-phenyloxazol-2-yl)picolinamide (11b)**

Light brown solid. Yield 36%. mp = 107–109°C. ^1H NMR (500 MHz, DMSO) δ 11.56 (s, 1H), 8.76 (dd, *J* = 4.7, 1.7 Hz, 1H), 8.51 (s, 1H), 8.16 (d, *J* = 7.7 Hz, 1H), 8.10 (td, *J* = 7.7, 1.7 Hz, 1H), 7.77 (s, 2H), 7.73 (dd, *J* = 7.7, 4.7 Hz, 1H), 7.45 (t, *J* = 7.7 Hz, 2H), 7.38 – 7.31 (m, 1H). ^{13}C NMR (126 MHz, DMSO) δ 163.14, 152.97, 149.01, 148.59, 139.74, 138.45, 132.70, 130.94, 129.00, 128.23, 127.92, 125.21, 123.11. IR (ATR-Ge, cm^{-1}) 3351, 3165, 1708 (*υ*NHCO), 1591, 1570, 1537. HPLC purity 99.9%. HRMS (HESI+) m/z = 266.0919 (theoretical for $M+H^+$ m/z = 266.0924, error –1.72 ppm).

***N*-(4-phenylthiazol-2-yl)nicotinamide (12a)**

CAS Registry Number 14397-15-0. Light beige solid. Yield 31%. mp = 228–231°C [lit. 223–224°C in EtOH[10]]. ^1H NMR (600 MHz, DMSO) δ 12.97 (s, 1H), 9.21 (dd, *J* = 2.3, 0.9 Hz, 1H), 8.76 (dd, *J* = 4.8, 1.6 Hz, 1H), 8.42 (ddd, *J* = 8.0, 2.3, 1.6 Hz, 1H), 7.94 – 7.89 (m, 2H), 7.69 (s, 1H), 7.55 (ddd, *J* = 8.0, 4.8, 0.9 Hz, 1H), 7.44 – 7.38 (m, 2H), 7.33 – 7.26 (m, 1H). ^{13}C (151 MHz, DMSO) δ 164.55, 158.75, 153.47, 149.75, 136.45, 134.81, 129.31, 128.55, 128.42, 126.33, 124.12, 109.33. IR (ATR-Ge, cm^{-1}) 3107, 2927, 1677 (*υ*NHCO), 1602, 1587, 1569, 1446. HPLC purity 99.9%. HRMS (HESI+) m/z = 282.0693 (theoretical for $M+H^+$ m/z = 282.0696, error –1.04 ppm).

***N*-(4-phenyloxazol-2-yl)nicotinamide (12b)**

Light yellow solid. Yield 12%. mp = 223.6°C. ^1H NMR (500 MHz, DMSO) δ 11.99 (s, 1H), 9.14 (d, *J* = 2.2 Hz, 1H), 8.80 (d, *J* = 4.8 Hz, 1H), 8.50 (s, 1H), 8.34 (dt, *J* = 7.9, 2.2 Hz, 1H), 7.78 (d, *J* = 7.5 Hz, 2H), 7.59 (dd, *J* = 7.9, 4.8 Hz, 1H), 7.45 (t, *J* = 7.5 Hz, 1H), 7.34 (t, *J* = 7.5 Hz, 1H). ^{13}C NMR (126 MHz, DMSO) δ 163.77, 153.36, 152.99, 149.10, 139.24, 135.92, 132.09, 130.73, 128.85, 128.66, 128.08, 125.01, 123.64. IR (ATR-Ge, cm^{-1}) 3058, 1697 (*υ*NHCO), 1619, 1594, 1449, 1429. HPLC purity 99.3%. HRMS (HESI+) m/z =266.0921 (theoretical for $M+H^+$ m/z = 266.0924, error –1.23 ppm).

***N*-(4-phenylthiazol-2-yl)isonicotinamide (13a)**

CAS Registry Number 14397-16-1. Light yellow solid. Yield 8%. mp = 207–209°C [lit. 206–208°C[9], 225 - 227°C[11], 213 - 216°C[12]]. ^1H NMR (600 MHz, $CDCl_3$) δ 11.74 (s, 1H), 8.59 (d, *J* = 5.0 Hz, 2H), 7.64 (d, *J* = 7.5 Hz, 2H), 7.53 (d, *J* = 5.0 Hz, 2H), 7.27 (t, *J* = 7.5 Hz, 2H), 7.24 – 7.19 (m, 2H). ^{13}C NMR (151 MHz, CDCl3) δ 163.94, 159.03, 150.69, 150.50, 139.22, 133.89, 128.89, 128.40, 126.19, 121.03, 108.76. IR (ATR-Ge, cm^{-1}) 2927, 1677 (*υ*NHCO), 1577, 1558, 1446, 1415. HPLC purity 99.9%. HRMS (HESI+) m/z =282.0693 (theoretical for $M+H^+$ m/z = 282.0696, error –1.04 ppm).

***N*-(4-phenyloxazol-2-yl)isonicotinamide (13b)**

Light yellow solid. Yield 32%. mp = 168–171°C. ^1H NMR (500 MHz, DMSO) δ 12.07 (s, 1H), 8.85 – 8.79 (m, 2H), 8.51 (s, 1H), 7.92 – 7.87 (m, 2H), 7.81 – 7.75 (m, 2H), 7.45 (t, J = 7.9 Hz, 2H), 7.38 – 7.31 (m, 1H). ^{13}C (126 MHz, DMSO) δ 163.72, 153.14, 150.48, 139.88, 139.32, 132.19, 130.68, 128.86, 128.11, 125.02, 121.76. IR (ATR-Ge, cm^{-1}) 3151, 1704 (*υ*NHCO), 1622, 1603, 1572, 1476, 1448. HPLC purity 99.9%. HRMS (HESI+) m/z =266.0921 (theoretical for $M+H^+$ m/z = 266.0924, error –1.23 ppm).

5-methyl-*N*-(4-phenyloxazol-2-yl)nicotinamide (14b)

White solid. Yield 27%. mp = 258.6°C. ^1H NMR (600 MHz, DMSO) δ 11.90 (s, 1H), 8.95 (s, 1H), 8.64 (s, 1H), 8.48 (s, 1H), 8.17 (s, 1H), 7.78 (d, *J* = 7.6 Hz, 2H), 7.45 (t, *J* = 7.6 Hz, 2H), 7.34 (t, *J* = 7.6 Hz, 1H), 2.39 (s, 3H). ^{13}C NMR (126 MHz, DMSO) δ 163.88, 153.30, 146.26, 139.26, 136.09, 133.08, 132.05, 130.73, 128.84, 128.22, 128.06, 125.00, 17.75. IR (ATR-Ge, cm^{-1}) 2926, 1691 (*υ*NHCO), 1615, 1592,

1516, 1489. HPLC purity 99.9%. HRMS (HESI+) m/z =280.1077 (theoretical for $M+H^+$ m/z = 280.1080, error –1.17 ppm).

2-chloro-*N*-(4-phenylthiazol-2-yl)isonicotinamide (15a)
CAS Registry Number 1043211-02-4. Light yellow solid. Yield 13%. mp = 158–162°C. ^1H NMR (500 MHz, DMSO) δ 13.16 (s, 1H), 8.64 (d, *J* = 5.1 Hz, 1H), 8.15 (d, *J* = 1.5 Hz, 1H), 8.00 (dd, *J* = 5.1, 1.5 Hz, 1H), 7.95 (d, *J* = 7.4 Hz, 2H), 7.76 (s, 1H), 7.45 (t, *J* = 7.4 Hz, 2H), 7.34 (t, *J* = 7.4 Hz, 1H). ^{13}C NMR (126 MHz, DMSO) δ 163.03, 158.30, 151.42, 151.30, 149.77, 143.22, 134.57, 129.26, 128.43, 126.25, 123.20, 121.89, 109.62. IR (ATR-Ge, cm^{-1}) 3076, 1680 (*υ*NHCO), 1545, 1470, 1445. HPLC purity 98.2%. HRMS (HESI+) m/z =316.0303 (theoretical for $M+H^+$ m/z = 316.0306, error –0.86 ppm).

2-chloro-*N*-(4-phenyloxazol-2-yl)isonicotinamide (15b)
Yellow solid. Yield 7%. mp = (221.9°C carbonization). ^1H NMR (600 MHz, ACETONE) δ 10.89 (s, 1H), 8.60 (d, *J* = 5.0 Hz, 1H), 8.21 (s, 1H), 8.00 (d, *J* = 1.5 Hz, 1H), 7.93 (dd, *J* = 5.0, 1.5 Hz, 1H), 7.82 – 7.73 (m, 2H), 7.40 (t, *J* = 7.5 Hz, 2H), 7.32 (t, *J* = 7.5 Hz, 1H). ^{13}C NMR (151 MHz, ACETONE) δ 151.88, 150.87, 128.78, 128.24, 125.33, 122.69, 121.07. IR (ATR-Ge, cm^{-1}) 3149, 1618 (*υ*NHCO), 1591, 1570, 1537. HRMS (HESI+) m/z =300.0531 (theoretical for $M+H^+$ m/z = 300.0534, error –1.11 ppm).

2-chloro-6-methyl-*N*-(4-phenylthiazol-2-yl)isonicotinamide (16a)
Light yellow solid. Yield 16%. mp = 92–96°C. ^1H (600 MHz, DMSO) δ 13.03 (s, 1H), 7.92 – 7.89 (m, 3H), 7.85 (d, *J* = 1.3 Hz, 1H), 7.71 (s, 1H), 7.41 (t, *J* = 7.6 Hz, 2H), 7.31 (t, *J* = 7.6 Hz, 1H), 2.52 (s, 3H). ^{13}C NMR (151 MHz, DMSO) δ 163.26, 160.79, 158.36, 150.52, 149.89, 143.44, 134.69, 129.32, 128.49, 126.32, 121.37, 120.12, 109.65, 24.22. IR (ATR-Ge, cm^{-1}) 3067, 1679 (*υ*NHCO), 1544, 1483, 1445, 1392, 1303. HPLC purity 99.9%. HRMS (HESI+) m/z =330.0458 (theoretical for $M+H^+$ m/z = 330.0462, error –1.21 ppm).

2-chloro-6-methyl-*N*-(4-phenyloxazol-2-yl)isonicotinamide (16b)
CAS Registry Number 457940-33-9. Yellow solid. Yield 8%. mp = 83–86°C. 1H NMR (600 MHz, $CDCl_3$) δ 12.52 – 10.44 (m, 1H), 7.63 (s, 1H), 7.57 (s, 1H), 7.49 (s, 1H), 7.45 – 7.39 (m, 2H), 7.29 – 7.23 (m, 3H), 2.39 (s, 3H). 13C NMR (151 MHz, $CDCl_3$) δ 166.48, 160.41, 157.20, 151.32, 144.49, 136.65, 129.37, 129.06, 129.00, 128.15, 125.44, 120.27, 119.88, 24.12. IR (ATR-Ge, cm^{-1}) 3141, 2924, 1708 (*υ*NHCO), 1622, 1551, 1489, 1402, 1293. HPLC purity 98.8%. HRMS (HESI+) m/z =314.0687 (theoretical for $M+H^+$ m/z = 314.0691, error –1.19 ppm).

***N*-(4-phenyloxazol-2-yl)pyrazine-2-carboxamide (17b)**
CAS Registry Number 2248961-45-5. Yellow solid. Yield 9%. mp = 140–142°C. ^1H NMR (600 MHz, DMSO) δ 11.78 (s, 1H), 9.25 (d, *J* = 1.5 Hz, 1H), 8.93 (d, *J* = 2.5 Hz, 1H), 8.79 (dd, *J* = 2.5, 1.5 Hz, 1H), 8.48 (s, 1H), 7.77 – 7.71 (m, 2H), 7.44 – 7.37 (m, 2H), 7.33 – 7.26 (m, 1H). ^{13}C NMR (151 MHz, DMSO) δ 162.81, 154.71, 148.91, 144.87, 144.43, 144.11, 140.14, 133.15, 131.24, 129.36, 129.00, 128.66, 125.58. IR (ATR-Ge, cm^{-1}) 3343, 3125, 2923, 2852, 1713 (*υ*NHCO), 1568, 1550, 1472, 1462. HPLC purity 95.0%. HRMS (HESI+) m/z = 267.0873 (theoretical for $M+H^+$ m/z = 267.0876, error –1.48 ppm).

5-chloro-*N*-(4-phenyloxazol-2-yl)pyrazine-2-carboxamide (18b)
CAS Registry Number 1699172-80-9. Light brown solid. Yield 6%. mp = 203–207°C. ^1H NMR (600 MHz, DMSO) δ 11.86 (s, 1H), 9.08 (d, *J* = 1.4 Hz, 1H), 8.93 (d, *J* = 1.4 Hz, 1H), 8.48 (s, 1H), 7.74 (d, *J* = 7.6 Hz, 2H), 7.41 (t, *J* = 7.6 Hz, 2H), 7.31 (t, *J* = 7.6 Hz, 1H). ^{13}C NMR (151 MHz, DMSO) δ 162.05, 152.99, 152.11, 144.88, 143.93, 143.18, 140.15, 133.22, 131.20, 129.36, 128.64, 125.57. IR (ATR-Ge, cm^{-1}) 3357, 2925,

2853, 1732 (υNHCO), 1616, 1567, 1530, 1456. HPLC purity 98.0%. HRMS (HESI+) m/z = 301.0483 (theoretical for M+H$^+$ m/z = 301.0487, error –1.27 ppm).

***N*-(4-phenylthiazol-2-yl)quinoxaline-2-carboxamide (19a)**

CAS Registry Number 920708-73-2. Yellow solid. Yield 6%. mp = 217– 219°C. ^{1}H NMR (600 MHz, DMSO) δ 12.65 (s, 1H), 9.52 (s, 1H), 8.35 – 8.21 (m, 1H), 8.21 – 8.10 (m, 1H), 8.04 – 7.97 (m, 2H), 7.96 – 7.85 (m, 2H), 7.76 (s, 1H), 7.42 (t, *J* = 7.4 Hz, 2H), 7.31 (t, *J* = 7.4 Hz, 1H). ^{13}C NMR (151 MHz, DMSO) δ 163.02, 157.94, 149.96, 144.40, 143.70, 140.44, 134.69, 133.13, 132.07, 130.36, 129.64, 129.30, 128.50, 126.40, 109.69. IR (ATR-Ge, cm^{-1}) 3359, 3089, 2933, 1689 (υNHCO), 1568, 1471, 1446, 1408, 1367. HPLC purity 99.6%. HRMS (HESI+) m/z =333.0798 (theoretical for M+H$^+$ m/z = 333.0805, error –1.90 ppm).

***N*-(4-phenyloxazol-2-yl)quinoxaline-2-carboxamide (19b)**

Dark yellow solid. Yield 15%. mp = 181–183°C. ^{1}H NMR (600 MHz, DMSO) δ 11.97 (s, 1H), 9.49 (s, 1H), 8.50 (s, 1H), 8.28 – 8.22 (m, 1H), 8.22 – 8.16 (m, 1H), 8.04 – 7.96 (m, 2H), 7.76 (d, *J* = 7.6 Hz, 2H), 7.42 (t, *J* = 7.6 Hz, 2H), 7.31 (t, *J* = 7.6 Hz, 1H). ^{13}C NMR (151 MHz, DMSO) δ 162.82, 153.23, 144.38, 143.84, 143.72, 140.26, 140.12, 133.14, 133.02, 132.10, 131.24, 130.27, 129.64, 129.38, 128.65, 125.59. IR (ATR-Ge, cm^{-1}) 3366, 3320, 31237, 2924, 1711 (υNHCO), 1618, 1569, 1540, 1471. HPLC purity 98.8%. HRMS (HESI+) m/z =317.1030 (theoretical for M+H$^+$ m/z = 317.1033, error –1.09 ppm).

***N*-(4-phenyloxazol-2-yl)benzamide (20b)**

CAS Registry Number 858017-35-3. Light yellow solid. Yield 21%. mp = 143–145°C. ^{1}H NMR (600 MHz, DMSO) δ 11.65 (s, 1H), 8.45 (s, 1H), 8.00 – 7.95 (m, 2H), 7.77 – 7.72 (m, 2H), 7.66 – 7.55 (m, 1H), 7.51 (t, *J* = 7.6 Hz, 2H), 7.45 – 7.38 (m, 2H), 7.33 – 7.27 (m, 1H). ^{13}C NMR (151 MHz, DMSO) δ 165.70, 154.15, 139.84, 133.24, 133.15, 132.67, 131.37, 129.36, 129.13, 128.66, 128.55, 125.53. IR (ATR-Ge, cm^{-1}) 3134, 2928, 1694 (υNHCO), 1601, 1570, 1450. HPLC purity 98.6%. HRMS (HESI+) m/z = 265.0968 (theoretical for M+H$^+$ m/z = 265.0971, error –1.42 ppm).

2.1.1. Representative NMR spectra

Compound 6b

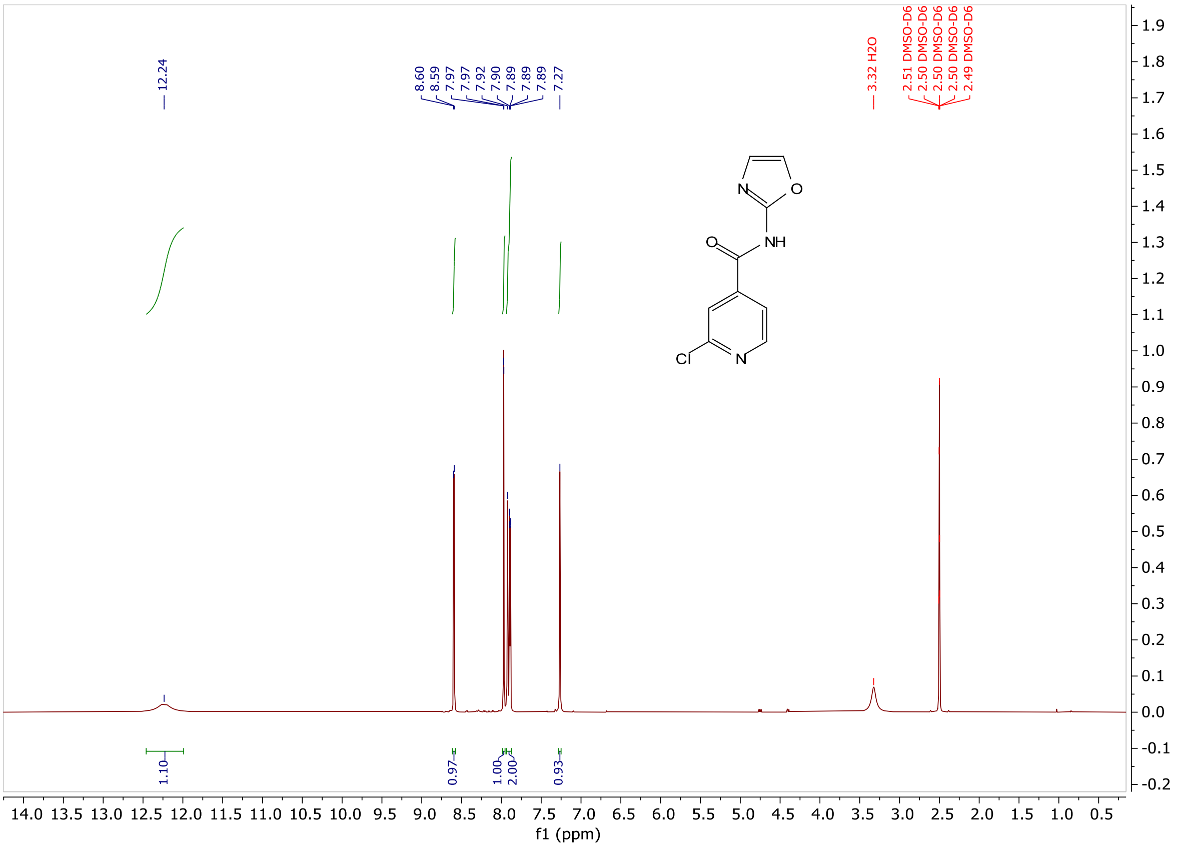

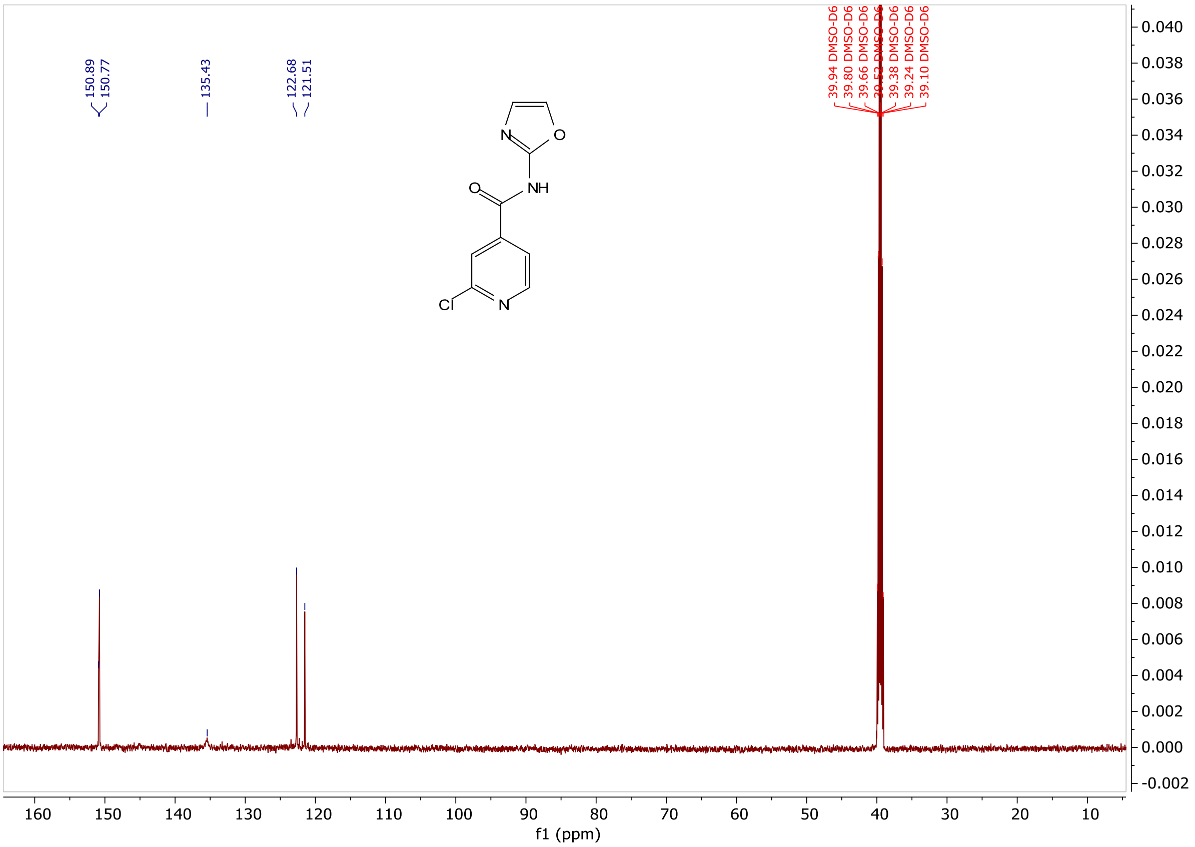

Compound 15b

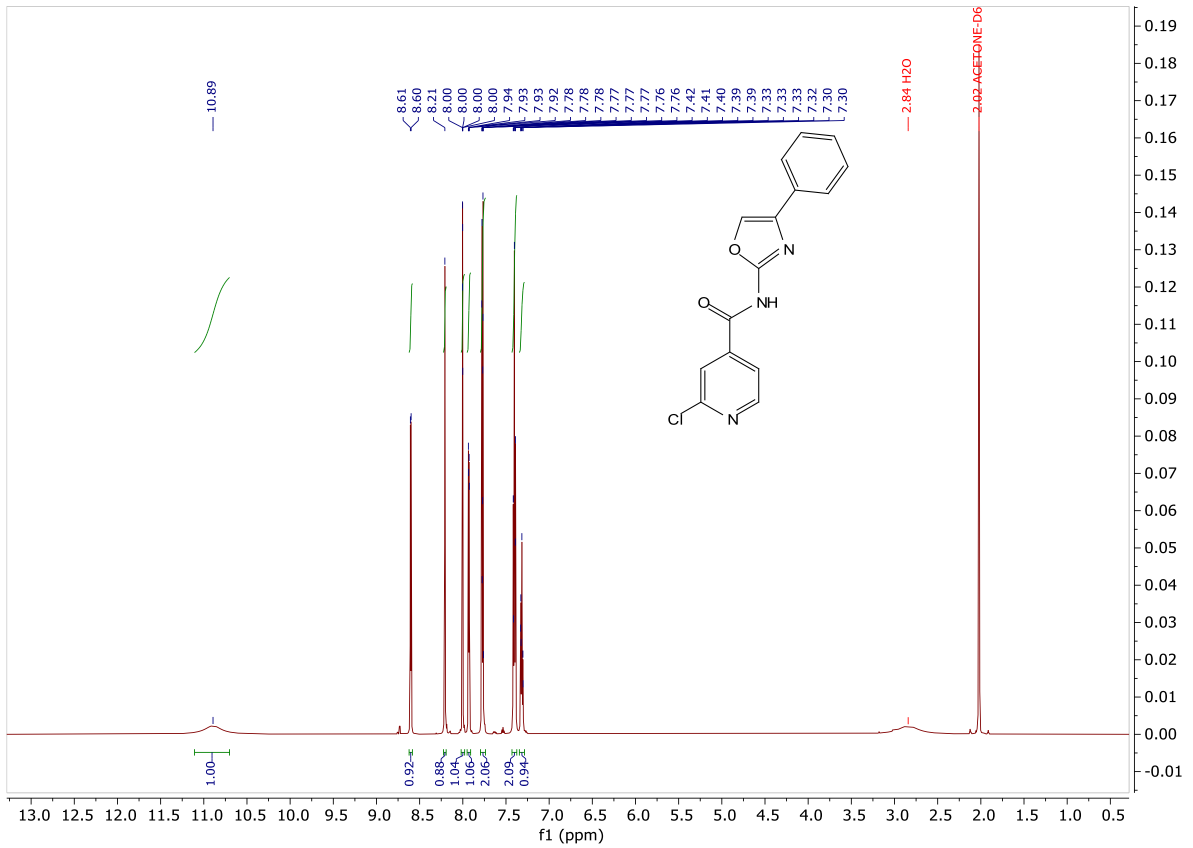

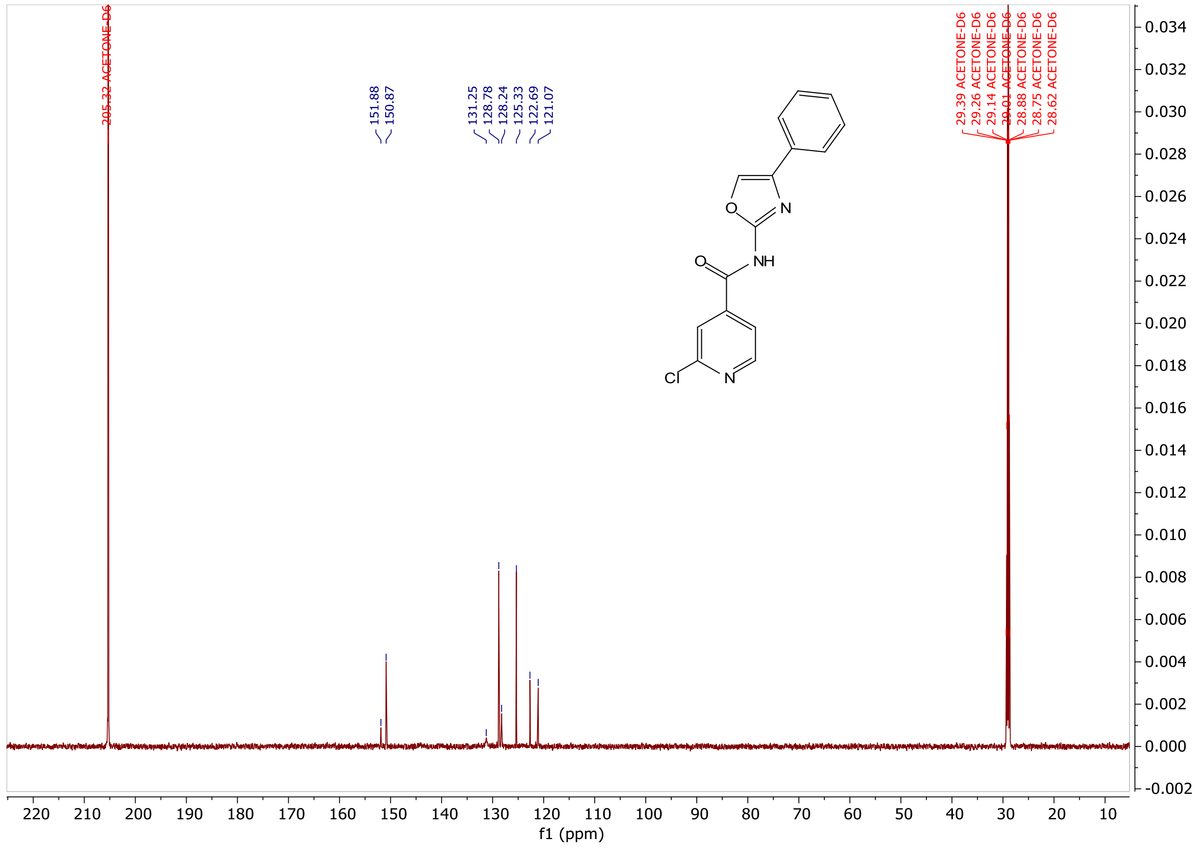

2.2. Investigation of lipophilicity

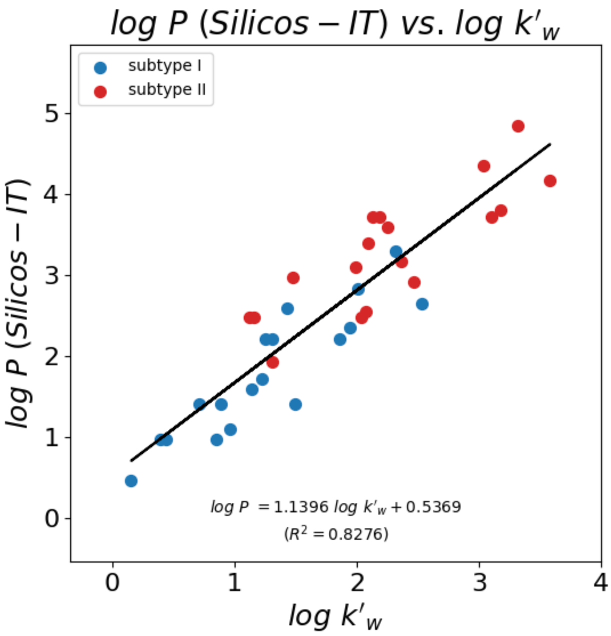

Figure S1. *Correlation plot of log P (calculated by SILICOS-IT) and log k'$_w$.*

2.3. *In silico* studies

2.3.1. Docking

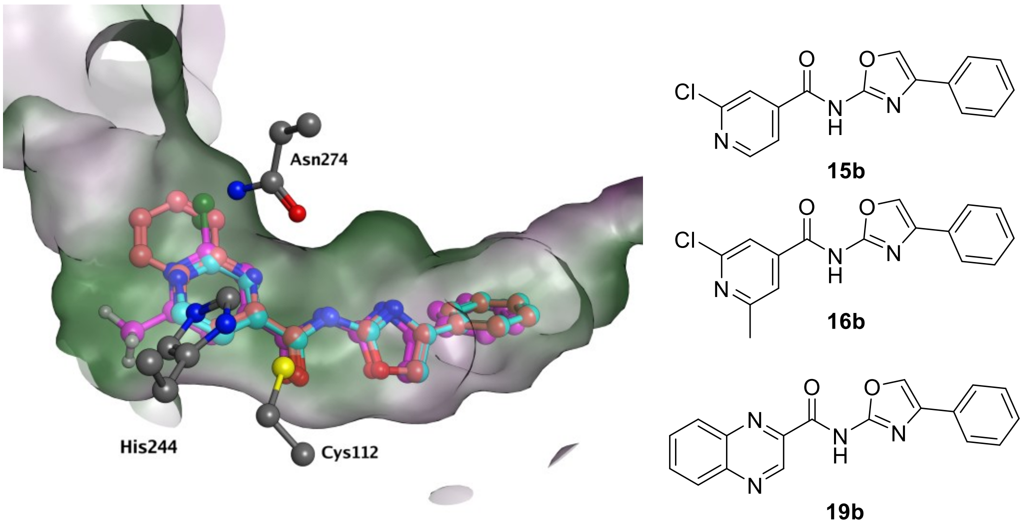

Figure S2. *Docked poses of* ***15b*** *(cyan),* ***16b*** *(magenta) and* ***19b*** *(pink) in MtFabH (PDB ID: 1U6S).*

2.3.2. Investigation of binding mode stability

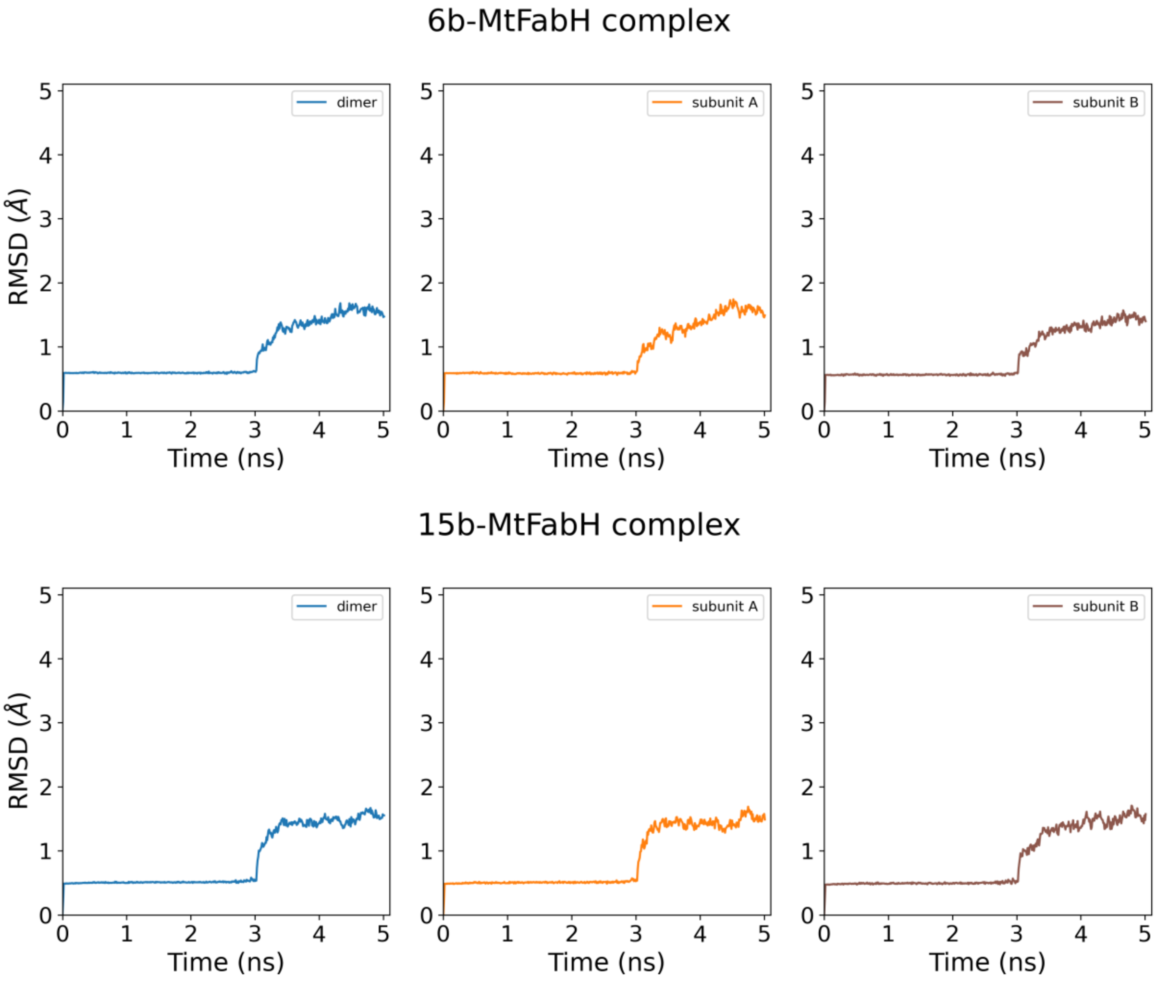

Figure S3. *Backbone RMSD of the MD minimisation-heating-equilibration stages of* ***6b****-MtFabH (top) and* ***15b****-MtFabH (bottom) complexes. Trajectory was superposed on the backbone atoms.*

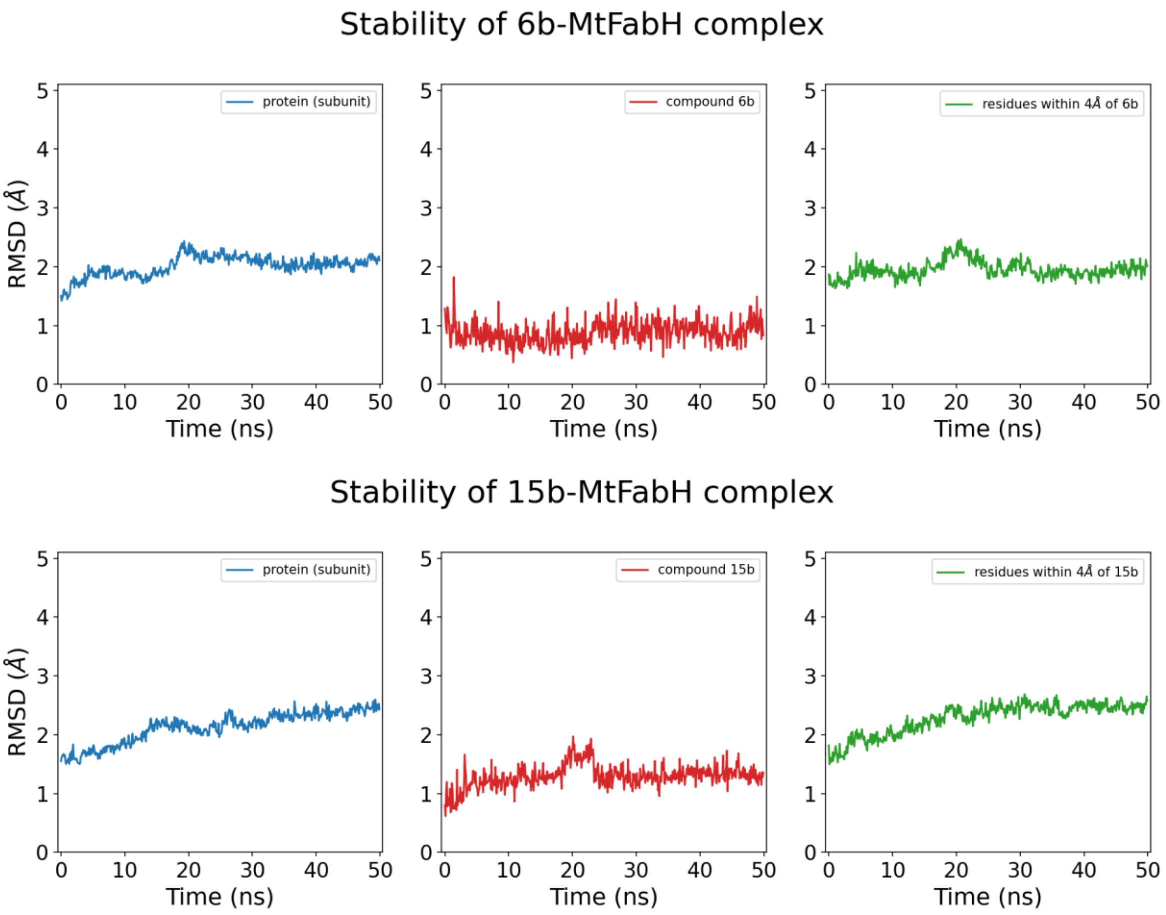

Figure S4. *Representative RMSD curves for the production runs of the derivatives* ***6b*** *(top) and* ***15b*** *(bottom). Trajectory was superposed on the respective subunit backbone.*

2.4. In vitro screening of antimycobacterial activity

Table S2. *Antimycobacterial activity against fast-growing and atypical mycobacteria. MICs are expressed in µg/mL.*

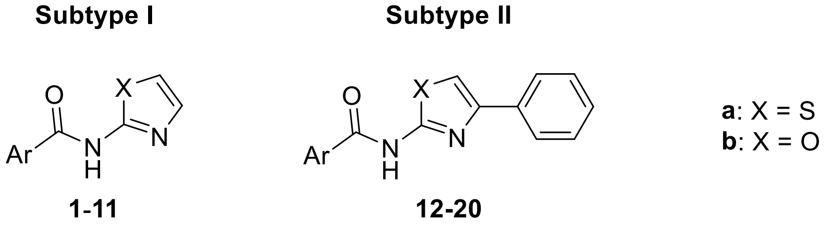

Compound			MIC (µg/mL)			
**Code**	**Ar**	**X**	***M. smegmatis***	***M. aurum***	***M. avium***	***M. kansasii***
**1a**	pyridin-2-yl	S	62.5	7.81	n.d.	n.d.
**1b**	pyridin-2-yl	O	125	31.25	n.d.	n.d.
**2a**	pyridin-3-yl	S	≥500	250	n.d.	n.d.
**2b**	pyridin-3-yl	O	62.5	15.625	n.d.	n.d.
**3a**	pyridin-4-yl	S	≥500	≥500	≥500	250
**3b**	pyridin-4-yl	O	62.5	15.625	n.d.	n.d.
**4b**	5-Me-pyridin-3-yl	O	≥500	31.25	125	62.5
**5b**	2-Me-pyridin-4-yl	O	250	15.625	62.5	31.25
**6a**	2-Cl-pyridin-4-yl	S	≥500	≥500	≥500	≥500
**6b**	2-Cl-pyridin-4-yl	O	6.25	3.125	3.125	3.125
**7a**	2-Cl-6-Me-pyridin-4-yl	S	≥500	≥500	≥500	≥500
**7b**	2-Cl-6-Me-pyridin-4-yl	O	7.81	3.91	3.91	7.81
**8a**	pyrazin-2-yl	S	≥500	125	250	31.25
**8b**	pyrazin-2-yl	O	≥500	31.25	62.5	15.625
**9a**	5-Cl-pyrazin-2-yl	S	≥500	≥500	15.625	<3.91
**9b**	5-Cl-pyrazin-2-yl	O	62.5	15.625	31.25	<3.91
**10a**	quinoxalin-2-yl	S	≥250	≥250	≥250	7.81
**10b**	quinoxalin-2-yl	O	62.5	15.625	n.d.	n.d.
**11a**	2-pyridyl	S	≥500	≥500	≥500	3.91
**11b**	2-pyridyl	O	125	31.25	31.25	3.91
**12a**	3-pyridyl	S	≥500	≥500	≥500	≥500
**12b**	3-pyridyl	O	500	250	n.d.	n.d.
**13a**	4-pyridyl	S	≥500	≥500	≥500	7.81
**13b**	4-pyridyl	O	125	31.25	n.d.	n.d.
**14b**	5-Me-pyridin-3-yl	O	≥250	≥250	≥250	≥250
**15a**	2-Cl-pyridin-4-yl	S	15.625	31.25	15.625	<3.91
**15b**	2-Cl-pyridin-4-yl	O	31.25	7.81	15.625	15.625
**16a**	2-Cl-6-Me-pyridin-4-yl	S	7.81	15.625	7.81	3.91
**16b**	2-Cl-6-Me-pyridin-4-yl	O	31.25	7.81	7.81	15.625
**17a**	pyrazin-2-yl	S	>250	n.d.	n.d.	>50
**17b**	pyrazin-2-yl	O	≥500	125	≥500	15.625
**18a**	5-Cl-pyrazin-2-yl	S	>500	n.d.	>100	>100
**18b**	5-Cl-pyrazin-2-yl	O	≥500	125	62.5	15.625
**19a**	quinoxalin-2-yl	S	≥500	≥500	≥500	≥500
**19b**	quinoxalin-2-yl	O	≥500	≥500	7.81	3.91
**20b**	phenyl	O	125	31.25	31.25	31.25
**INH**	-	-	15.625–31.25	3.91	1000	3.125–6.25
**RIF**	-	-	12.5	0.39	0.0625-0.03125	0.025
**CIP**	-	-	0.125	0.015625	0.25-0.5	0.25

n.d. – not determined; INH – isoniazid; RIF – rifampicin; CIP – ciprofloxacin

2.5. In vitro screening of antibacterial activity

Abbreviation	Full name
SA	*Staphylococcus aureus* subsp. *aureus* CCM 4223 (ATCC 29213)
MRSA	*Staphylococcus aureus* subsp. *aureus* (MRSA) CCM 4750 (ATCC 43300)
SE	*Staphylococcus epidermidis* CCM 4418 (ATCC 12228)
EF	*Enterococcus faecalis* CCM 4224 (ATCC 29212)
EC	*Escherichia coli* CCM 3954 (ATCC 25922)
KP	*Klebsiella pneumoniae* CCM 4415 (ATCC 10031)
AB	*Acinetobacter baumannii* DSM 30007 (ATCC 19606)
PA	*Pseudomonas aeruginosa* CCM 3955 (ATCC 27853)

Table S3. *Antibacterial activity against tested bacterial species after 24h incubation (72h for PA). MICs are expressed in µM.*

	MIC (µM)							
**Code**	**SA**	**MRSA**	**SE**	**EF**	**EC**	**KP**	**AB**	**PA**
**1a**	>500	>500	>500	>500	>500	>500	>500	>500
**1b**	125	250	125	>500	>500	>500	>500	>500
**2a**	>500	>500	>500	>500	>500	>500	>500	>500
**2b**	250	500	500	>500	>500	>500	>500	>500
**3a**	>500	>500	>500	>500	>500	>500	>500	>500
**3b**	250	500	500	>500	>500	>500	>500	>500
**4b**	500	500	500	>500	>500	500	500	>500
**5b**	>500	>500	>500	>500	>500	>500	>500	>500
**6a**	>500	>500	>500	>500	>500	>500	>500	>500
**6b**	250	62.5	62.5	500	62.5	125	62.5	>500
**7a**	>500	>500	>500	>500	>500	>500	>500	>500
**7b**	62.5	125	125	500	250	125	500	>500
**8a**	>500	>500	500	>500	>500	>500	>500	>500
**8b**	>500	>500	>500	>500	>500	>500	>500	>500
**9a**	>500	>500	>500	>500	>500	>500	>500	>500
**9b**	>500	>500	>500	>500	>500	>500	>500	>500
**10a**	>500	>500	>500	>500	>500	>500	>500	>500
**10b**	500	>500	500	>500	>500	>500	>500	>500
**11a**	>500	>500	>500	>500	>500	>500	>500	>500
**11b**	500	500	125	>500	>500	>500	>500	>500
**12a**	>125	>125	>125	>125	>125	>125	>125	>125
**12b**	500	>500	62.5	>500	>500	>500	>500	>500
**13a**	>125	>125	>125	>125	>125	>125	>125	>125
**13b**	500	>500	125	>500	>500	>500	>500	>500
**14b**	250	250	250	>250	>250	>250	>250	>250
**15a**	>500	>500	>500	>500	>500	>500	>500	>500
**15b**	62.5	62.5	250	250	>500	500	500	>500
**16a**	>500	>500	>500	>500	>500	>500	>500	>500
**16b**	62.5	62.5	125	>125	>125	>125	>125	>125
**17a**	insoluble	insoluble	insoluble	insoluble	insoluble	insoluble	insoluble	insoluble
**17b**	>500	>500	>500	>500	>500	>500	>500	>500
**18a**	insoluble	insoluble	insoluble	insoluble	insoluble	insoluble	insoluble	insoluble
**18b**	insoluble	insoluble	insoluble	insoluble	insoluble	insoluble	insoluble	insoluble
**19a**	>250	>250	>250	>250	>250	>250	>250	>250
**19b**	>500	>500	>500	>500	>500	>500	>500	>500
**20b**	250	500	250	>500	>500	>500	500	>500

For MIC of standards see section 1.1.3. Antibacterial screening.

2.6. In vitro screening of antifungal activity

Abbreviation	Full name
CA	*Candida albicans* CCM 8320 (ATCC 24433)
CK	*Candida krusei* CCM 8271 (ATCC 6258)
CP	*Candida parapsilosis* CCM 8260 (ATCC 22019)
CT	*Candida tropicalis* CCM 8264 (ATCC 750)
AF	*Aspergillus fumigatus* (ATCC 204305)
AFla	*Aspergillus flavus* CCM 8363
LC	*Lichtheimia corymbifera* CCM 8077
TI	*Trichophyton interdigitale* CCM 8377 (ATCC 9533)

Table S4. *Antifungal activity against tested fungal species after 24h incubation (72h for TI). MICs are expressed in µM.*

	**MIC (µM)**							
**Code**	**CA**	**CK**	**CP**	**CT**	**AF**	**AFla**	**LC**	**TI**
**1a**	>500	500	500	>500	500	>500	>500	500
**1b**	>500	>500	>500	>500	>500	>500	>500	>500
**2a**	>500	>500	>500	>500	>500	>500	>500	>500
**2b**	500	500	250	>500	>500	>500	>500	125
**3a**	>500	>500	>500	>500	>500	>500	>500	>500
**3b**	500	500	>500	>500	>500	>500	500	500
**4b**	>500	>500	>500	>500	>500	>500	>500	>500
**5b**	>125	>125	>125	>125	>125	>125	>125	>125
**6a**	>500	>500	>500	>500	>500	>500	>500	>500
**6b**	31.25	250	250	500	125	500	62.5	125
**7a**	>125	>125	>125	>125	>125	>125	>125	>125
**7b**	125	500	500	>500	250	>500	125	125
**8a**	>500	>500	>500	>500	>500	>500	>500	>500
**8b**	>500	>500	>500	>500	>500	>500	500	500
**9a**	>500	>500	>500	>500	500	500	>500	>500
**9b**	250	250	500	>500	500	>500	500	62.5
**10a**	>500	>500	>500	>500	>500	>500	>500	>500
**10b**	500	250	500	500	500	>500	500	250
**11a**	>500	>500	>500	>500	>500	>500	>500	>500
**11b**	500	500	500	500	500	500	125	62.5
**12a**	>125	>125	>125	>125	>125	>125	>125	>125
**12b**	>500	>500	>500	>500	>500	>500	>500	>500
**13a**	>125	>125	>125	>125	>125	>125	>125	>125
**13b**	>500	>500	>500	>500	>500	>500	>500	500
**14b**	>500	>500	>500	>500	>500	>500	>500	>500
**15a**	>125	>125	>125	>125	>125	>125	>125	>125
**15b**	500	500	500	500	>500	>500	>500	500
**16a**	>125	>125	>125	>125	>125	>125	>125	>125
**16b**	>125	>125	>125	>125	>125	>125	>125	62.5
**17a**	insoluble	insoluble	insoluble	insoluble	insoluble	insoluble	insoluble	insoluble
**17b**	>500	>500	>500	>500	>500	>500	>500	>500
**18a**	insoluble	insoluble	insoluble	insoluble	insoluble	insoluble	insoluble	insoluble
**18b**	insoluble	insoluble	insoluble	insoluble	insoluble	insoluble	insoluble	insoluble
**19a**	>125	>125	>125	>125	>125	>125	>125	>125
**19b**	>125	>125	>125	>125	>125	>125	>125	>125
**20b**	500	500	500	500	500	500	500	250

For MIC of standards see section *1.1.4. Antifungal screening*.

2.7. Cytotoxicity screening

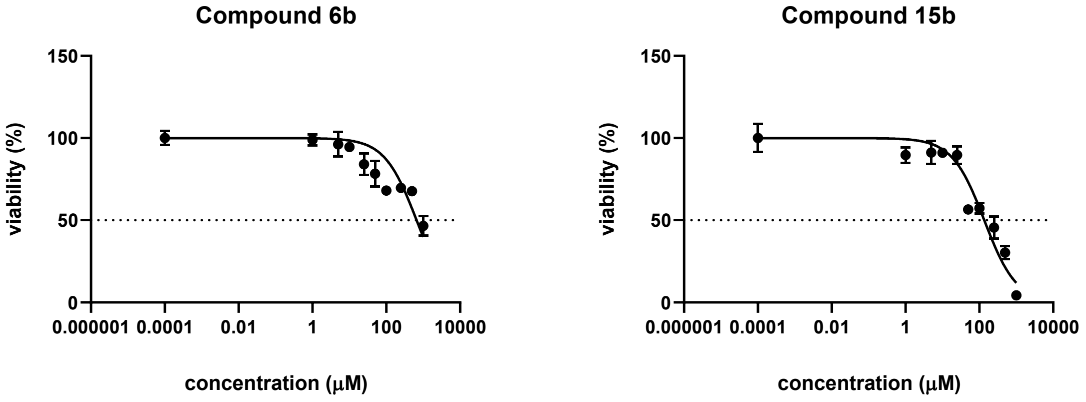

Figure S5. *Representative HepG2 cytotoxicity IC_{50} curves of the derivatives* ***6b*** *(left) and* ***15b*** *(right).*

3. Description of the supplied csv file

columns 1–3 Laboratory codes and manuscript IDs of compounds

columns 4–9 Descriptors calculated by ChemOffice20 (PerkinElmer Informatics)

columns 10–11 Experimental values of log k'_w and log S reported in the manuscript

columns 12–17 Descriptors calculated by eDragon (http://146.107.217.178/lab/edragon/start.html)

columns 18–62 Descriptors calculated by SwissADME (http://www.swissadme.ch/)

References

1. Franzblau, S.G.; Witzig, R.S.; McLaughlin, J.C.; Torres, P.; Madico, G.; Hernandez, A.; Degnan, M.T.; Cook, M.B.; Quenzer, V.K.; Ferguson, R.M.; et al. Rapid, low-technology MIC determination with clinical Mycobacterium tuberculosis isolates by using the microplate Alamar Blue assay. *J. Clin. Microbiol.* **1998**, *36*, 362-366, doi:10.1128/JCM.36.2.362-366.1998.
2. European Committee for Antimicrobial Susceptibility Testing (EUCAST) of the European Society of Clinical Microbiology and Infectious Diseases (ESCMID). Determination of minimum inhibitory concentrations (MICs) of antibacterial agents by broth dilution. *Clin. Microbiol. Infect.* **2003**, *9*, ix-xv, doi:10.1046/j.1469-0691.2003.00790.x.
3. EUCAST DEFINITIVE DOCUMENT E.DEF 7.3.1. Method for the determination of broth dilution minimum inhibitory concentrations of antifungal agents for yeasts. Available online: http://www.eucast.org/astoffungi/methodsinantifungalsusceptibilitytesting/susceptibility_testing_of_yeasts/ (accessed on 8 April, 2022).
4. EUCAST DEFINITIVE DOCUMENT E.DEF 9.3.1. Method for the determination of broth dilution minimum inhibitory concentrations of antifungal agents for conidia forming moulds. Available online: http://www.eucast.org/astoffungi/methodsinantifungalsusceptibilitytesting/susceptibility_testing_of_moulds/ (accessed on 8 April, 2022).
5. Modrić, M.; Božičević, M.; Faraho, I.; Bosnar, M.; Škorić, I. Design, synthesis and biological evaluation of new 1,3-thiazole derivatives as potential anti-inflammatory agents. *J. Mol. Struct.* **2021**, *1239*, 130526, doi:https://doi.org/10.1016/j.molstruc.2021.130526.
6. Simon, I.B. Synthesis of some derivatives of nicotinic acid. *Zh. Obshch. Khim.* **1951**, *21*, 1537-1540.
7. Clauder, O.; Toldy, L. Substances with tuberculostatic effects. III. *Magyar Kemiai Folyoirat* **1950**, *56*, 451-452
8. Takatori, K.; Imai, K.; Nakano, S.; Hasegawa, T.; Asano, S. Syntheses of the Amides Derived from Heterocyclic Acids and Heterocyclic Amines and Their Antitumor Activities. *Yakugaku Zasshi* **1976**, *96*, 471-474, doi:10.1248/yakushi1947.96.4_471.
9. Bilinski, S.; Urban, T. Synthesis of some derivatives of isonicotinic acid. I. *Annales Universitatis Mariae Curie-Sklodowska, Sectio DD: Medicina Veterinaria* **1597**, *12D*, 41-45.
10. Lanfranchi, F. Nicotinylaminothiazoles. I. 2-Nicotinylamino-4-methylthiazole and -4-phenylthiazole. *Atti. accad. Italia, Rend. Classe sci. fis. mat. nat.* **1942**, *3*, 776-781.
11. Gaddam, L.T.; Thata, S.; Adivireddy, P.; Venkatapuram, P. Synthesis of carboxamide and sulfonyl carboxamide linked heterocycles under green conditions. *ARKIVOC* **2019**, *2019*, 43-54, doi:10.24820/ark.5550190.p010.820.
12. Zhang, W.-T.; Ruan, J.-L.; Wu, P.-F.; Jiang, F.-C.; Zhang, L.N.; Fang, W.; Chen, X.-L.; Wang, Y.; Cao, B.-S.; Chen, G.-Y.; et al. Design, Synthesis, and Cytoprotective Effect of 2-Aminothiazole Analogues as Potent Poly(ADP-Ribose) Polymerase-1 Inhibitors. *J. Med. Chem.* **2009**, *52*, 718-725, doi:10.1021/jm800902t.
